# Identification of a novel minor-groove DNA binder that represses mitochondrial gene expression and induces apoptosis in highly aggressive leiomyosarcoma cells

**DOI:** 10.1038/s41420-025-02803-3

**Published:** 2025-11-10

**Authors:** Eleonora Malavasi, Raffaella Picco, Showmeya Mallavarapu, Martina Minisini, Francesca D’Este, Alessio Bertozzo, Lidia Giuliani, Roberta Astolfi, Monica Chinellato, Giacomo Bettin, Marco Bortoluzzi, Rino Ragno, Alessandro Angelini, Claudio Brancolini

**Affiliations:** 1 Department of Medicine, Lab of Epigenomics, Università degli Studi di Udine, Udine, 33100 Italy; 2 Rome Center for Molecular Design, Department of Drug Chemistry and Technology, Sapienza University of Rome, Piazzale Aldo Moro 5, 00185 Rome, Italy; 3 Department of Biology, University of Padua, Via U. Bassi 58, 35131 Padova, Italy; 4 Department of Molecular Sciences and Nanosystems, Ca’ Foscari University of Venice, Via Torino 155, 30172 Mestre, Italy; 5 European Centre for Living Technology (ECLT), Ca’ Bottacin, Dorsoduro 3911, Calle Crosera, 30123 Venice, Italy

**Keywords:** Virtual screening, Sarcoma, Apoptosis

## Abstract

Leiomyosarcoma (LMS) is an aggressive tumor for which there are few effective therapeutic options. Through a combination of in silico and in vitro screens, we have identified the compound NSC-260594/XMH95 as a promising molecule that selectively induces apoptosis in aggressive LMS cells by upregulating the BH3-only genes *PMAP1/NOXA, BIK, HRK* and *BBC3/PUMA*. Similar to the dye Hoechst 33258, XMH95 appears to bind the minor groove of DNA. Unlike Hoechst 33258, XMH95 converts to a fluorescent form only after DNA binding. Furthermore, unlike Hoechst 33258, XMH95 suppresses mitochondrial gene expression and is a more effective inducer of apoptosis. Apart from suppressing mitochondrial genes, XMH95 has many effects on gene expression that it shares with Hoechst 33258. By inhibiting mitochondrial transcription, we show that XMH95 induces apoptosis by impairing both nuclear and mitochondrial transcription. In summary, XMH95 is a novel DNA binder that triggers apoptosis by upregulating multiple BH3-only genes.

## Introduction

Leiomyosarcoma (LMS) is a highly aggressive and rare type of cancer that accounts for 10 to 20% of all soft tissue sarcomas [1, 2]. LMS originates from smooth muscle cells and can develop anywhere in the body, most commonly in the retroperitoneum, large blood vessels and uterus [3, 4]. Uterine LMS has an incidence of 0.64 cases per 100,000 women and shows some peculiarities compared to other LMSs [1, 2, 5]. In general, LMS shows a low mutational load and a complex karyotype with a high rate of aneuploidy [5, 6]. Mutations in the tumor suppressors *RB1*, *TP53, PTEN, CDKN2A, CDKN2B* and telomere-maintaining genes are common in LMS [5, 7]. These tumors are also immunologically cold, which limits the efficacy of immune checkpoint inhibitors [8, 9].

Since the early 1970s, doxorubicin monotherapy has been the standard first-line treatment for metastatic LMS. In recent decades, several studies have investigated different drug combinations, which have shown a general overall survival of around 20 months [10]. Trabectedin, a naturally derived drug that binds to the minor groove of DNA and interferes with transcription and DNA repair, has shown some efficacy in LMS patients [11, 12]. More recently, some improvement in overall survival and progression-free survival has been observed in patients with uterine or soft tissue leiomyosarcoma when doxorubicin was used in combination with trabectedin compared to doxorubicin alone [10].

Despite some partial improvements in the clinic, the identification of new potential pharmacological treatments for LMS is still an urgent unresolved problem. Many studies have explored new strategies to stop the proliferation of LMS cells and induce apoptosis [6]. For example, the efficacy of CDK and BCL2 inhibitors has been demonstrated in preclinical models [13–16].

The identification of new compounds that target alternative cellular activities could open new perspectives for LMS therapy. Following in silico and functional screening, we have identified a new compound that binds to the minor groove of DNA and induces apoptosis, particularly in highly proliferating LMS cells.

## Results

### Identification of new compounds with anti-proliferative activity against LMS cells

**Fig. 1:**
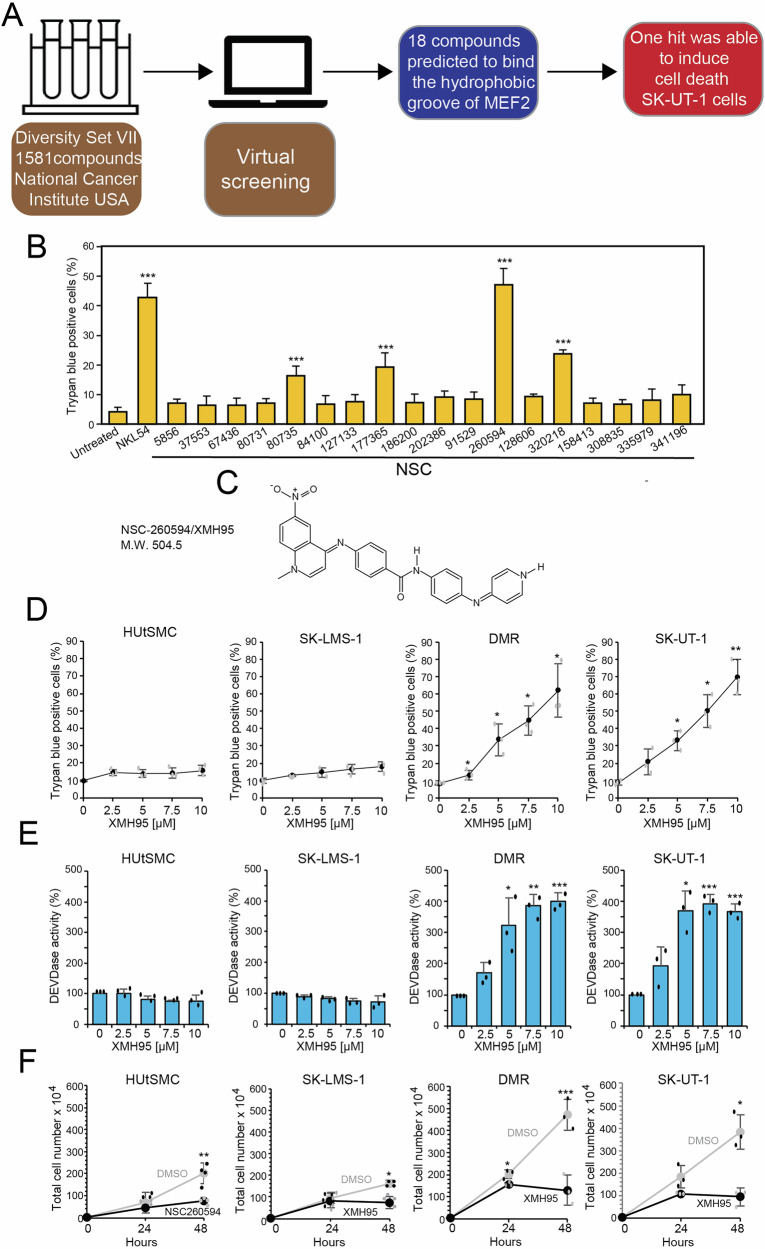
NSC-260594/XMH95 induces apoptosis in highly aggressive LMS cells. **A** Schematic description of the screening process adopted to isolate anti-proliferative compounds against LMS cells is provided. First, the compounds in the library were evaluated for their ability to fit into the MEF2 hydrophobic groove through computational analysis. The selected hits were then evaluated in parallel for their ability to trigger LMS cell death and compete with the HDAC4 peptide for binding to the MEF2D recombinant protein. **B** Cell death in SK-UT-1 cells after 48 hours of incubation with the indicated compounds. Cell death was calculated as a percentage of cells positive to Trypan blue staining. NKL54 was used as positive control (n = 3). ***p < 0.001, relative to DMSO-treated cells (Untreated). **C** Chemical structure of the compound NSC-260594/XMH95. **D** Cell death in the indicated LMS cell lines and in immortalized HUtSMC. Cells were treated with the indicated concentrations of XMH95. Cell death was calculated 48 hours later as the percentage of cells positive to Trypan blue staining. Data are presented as mean + SD (n = 3). *p < 0.05, **p < 0.01, ***p < 0.001, relative to DMSO-treated cells (**0**). **E** Caspase 3/7 activity of immortalized HUtSMC and the indicated LMS cell lines treated with different concentrations of XMH95. The DEVDase activity was measured after 36 hours. Data are presented as mean + SD (n = 3). *p < 0.05, **p < 0.01, ***p < 0.001, relative to DMSO-treated cells (0). **F** Cell proliferation assay of the indicated cell lines treated with 10 µM of XMH95 or DMSO alone. Analysis was performed at the indicated times and only Trypan blue-negative cells were counted. Data are presented as mean + SD (n = 3). *p < 0.05, **p < 0.01, ***p < 0.001, relative to DMSO-treated cells (0).

LMS is an aggressive cancer with few therapeutic options. To identify novel compounds that could affect LMS cells proliferation and survival through alternative mechanisms, we screened in silico compounds of Diversity Set VII from the Development Therapeutics Program of the National Cancer Institute (USA). The aim of the screening was to identify new small molecules capable of interfering with protein-protein interactions. Among several protein targets known to be dysregulated in LMS, we sought to identify compounds that might interfere with the activity of myocyte enhancer factor-2 (MEF2), a transcription factor that interacts with class IIa histone deacetylases (HDACs). Indeed, the MEF2 class IIa HDAC axis is altered in approximately 50% of LMS patients, and disruption of the functions of this axis affects the proliferation and survival of LMS cells [17, 18]. In particular, we focused on the search for small molecules that are able to accommodate themselves in the hydrophobic groove of MEF2, which serves as a binding site for various MEF2 partners, including class IIa HDACs [19]. We envisioned that the identified compounds act as interferers of protein-protein interactions. In silico screening of 1581 compounds revealed 18 small molecules predicted to bind to the hydrophobic groove of MEF2 (Fig. 1A and Table S1). These 18 compounds were subsequently evaluated for their ability to induce cell death in SK-UT-1 LMS cells and to abrogate the interaction between MEF2D and HDAC4. To achieve this goal, we took advantage of a fluorescently labelled HDAC4 peptide (fluo-pHDAC4) known to bind MEF2D and applied a fluorescence polarization-based assay recently developed by our group [19, 20] (Fig. S1A). The test showed that none of the 18 compounds was able to compete with fluo-pHDAC4 for binding to MEF2D (Fig. S1B-D). However, the compound NSC-260594, 4-[(1-methyl-6-nitroquinolin-4-ylidene)amino]-N-[4-[(1-methylpyridin-4-ylidene)amino]phenyl]benzamide, herein renamed XMH95, proved particularly efficient in killing LMS cells (Fig. 1B and C). As a positive control, we used NKL54, an HDAC1/2/3-specific inhibitor [20].

Although XMH95 failed to disrupt the interaction between MEF2 and class IIa HDACs, its potent cytotoxic activity prompted us to characterize its function in more detail. First, we investigated its proliferation inhibitory effect in a group of LMS cells compared to hTERT-immortalized human uterine smooth muscle cells (HUtSMC). Interestingly, XMH95 efficiently induced cell death in aggressive LMS cells (SK-UT-1 and DMR), but not in less aggressive (SK-LMS-1) and immortalized HUtSMC (Fig. 1D). This cell death proved to be apoptotic and was characterized by caspase activation (Fig. 1E). Although XMH95 did not induce apoptosis in less aggressive cells, it showed a cytostatic effect that restricted their proliferation (Fig. 1F).

To exclude the possibility that the differential effect of XMH95 on aggressive LMS cells and non-cancerous HUtSMC is due to different proliferation rates, we calculated the doubling times of the two cell lines. SK-UT-1 cells showed a doubling time of (19.87 +/- 4.2 hours), which was very similar to that of HUtSMC (18.73 +/- 9.47). Therefore, the induction of apoptosis cannot be due to differences in the proliferation rate of the two cell lines.

### The influence of XMH95 on cellular transcriptome

**Fig. 2:**
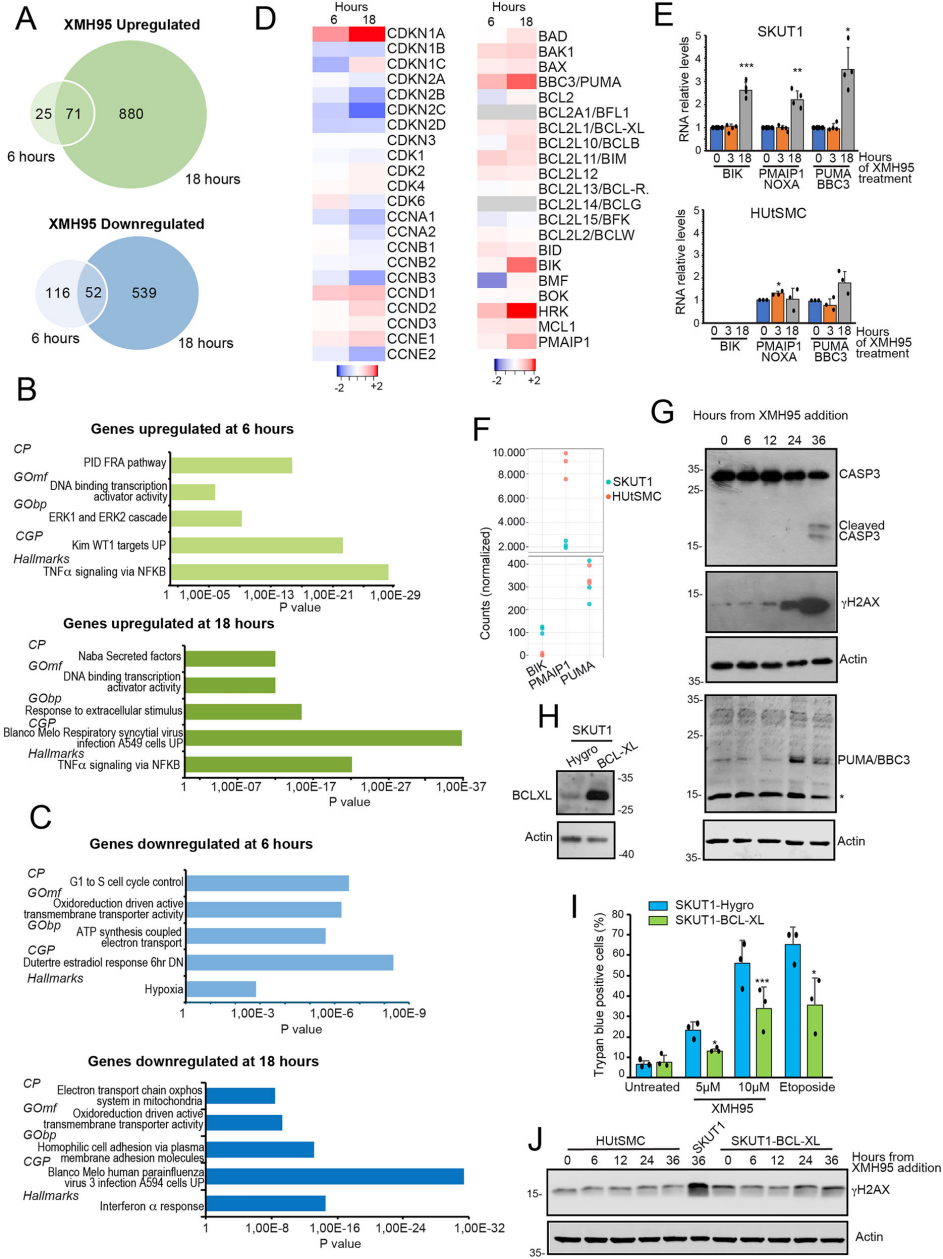
NSC-260594/XMH95 induces apoptosis in highly aggressive LMS cells. **A** Venn diagrams showing the number of transcripts upregulated in SK-UT-1 cells after 6 and 18 hours of treatment with 10 µM of XMH95. **B** Bar plots of functional enrichments using the GSEA and the Molecular Signatures Database (MSigDB) tools. The analysis was performed for genes upregulated after XMH95 treatment at 6 and 18 hours. Only the top terms are indicated. The detailed data are shown in Tables S3 and S4. **C** Bar plots of functional enrichments using the GSEA and the Molecular Signatures Database (MSigDB) tools. The analysis was performed for genes downregulated after XMH95 treatment at 6 and 18 hours. Only the top terms are indicated. The detailed data are shown in Tables S5 and S6. Gene Ontology (GO), Chemical and Genetic Perturbations (CGP), Canonical Pathways (CP). **D** Regulation of the cell cycle machinery in response to XMH95. Heatmaps reporting the expression levels (log2 fold change relative to untreated cells) of genes encoding for the cell cycle machinery (left) and BCL2 family members (right) in response to XMH95 at 6 and 18 hours of treatment. **E** Expression levels of the indicated BH3-only genes in HUtSMC and SK-UT-1 cells treated with 5 µM of XMH95. RNAs were extracted at the indicated times and processed for qRT-PCR. Data are relative to DMSO-treated cells. Mean ± SD; n = 3 or 4. *p < 0.05, **p < 0.01, ***p < 0.001 relative to DMSO-treated cells. **F** BIK, PMAIP1/NOXA and BBC3/PUMA expression in HUtSMC and SK-UT-1 cells. Expression values are shown as normalized counts obtained after DESeq2’s median of ratios normalization. **G** SK-UT-1 cells were treated for the indicated times with XMH95 [10 µM]. Cellular lysates were generated and immunoblot performed using the indicated antibodies. Actin was used as loading control. **H** Immunoblot analysis of SK-UT-1 cells engineered to express BCL-XL or Hygro as control by retroviral infection. Immunoblot was performed using the indicated antibodies. Actin was used as loading control. **I** Cell death in the SK-UT-1 cells expressing BCL-XL or Hygro genes. Cells were treated with the indicated concentrations of XMH95. Cell death was calculated 48 hours later as the percentage of cells positive to Trypan blue staining. Data are presented as mean ± SD (n = 3). Etoposide [5 µM] was used as control. *p < 0.05, **p < 0.01, ***p < 0.001, relative to the corresponding SKT-UT-1-Hygro cells (0). **J** HUtSMC and SK-UT-1/BCL-XL cells were treated for the indicated times with XMH95 [10 µM]. Cellular lysates were generated and immunoblot performed using the indicated antibodies. Actin was used as loading control. Lysates from SK-UT-1 cells treated for 36 with10µM of XMH95 were used as reference for γH2AX signal.

To gain insight into the mechanisms of action used by XMH95 to induce apoptosis, we performed RNA-seq experiments. To investigate the early response genes, we treated SK-UT-1 cells with XMH95 for 6 hours. To characterize late response genes, we extended treatment to 18 hours. Principal component analysis (PCA) showed the high reproducibility of the three biological replicates (Fig. S2A). The number of upregulated genes after 6 hours of treatment was 96 and increased to 952 after 18 hours (Fig. 2A and Table S2). Similarly, the number of downregulated genes increased from 168 to 591 after 6 and 18 hours (Fig. 2A and Table S2). Thus, XMH95 induced gene repression as the predominant response at early time points, whereas the late adaptive response was mainly characterized by an increase in gene expression.

To understand the cellular signaling pathways in which XMH95 is involved, we used the Molecular Signatures Database (MSigDB) [21, 22]. We first analyzed genes upregulated by treatment. The most highly enriched gene categories are shown (Fig. 2B and Table S3/S4). Early upregulated genes included several NF-kB and AP-1 targets, known to orchestrate inflammation (Fig. 2B and Table S3/S4). Genes upregulated by the Wilms tumor suppressor gene (WT1) were also upregulated in response to XMH95. Late responses involved instead inflammatory genes and extracellular matrix components. Transcription factors are an enriched GO category among both early and late response genes, indicating that XMH95 affects gene transcription. Early downregulated genes included genes related to the cell cycle, possibly reflecting growth arrest, and genes involved in respiration and ATP synthesis, which are still repressed 18 hours after treatment. At later time points, interferon and antiviral responses were highly enriched among the repressed genes (Fig. 2C and Table S5/S6).

### XMH95 engages the intrinsic/mitochondrial apoptotic pathway to induce cell death by upregulating multiple BH3-only proteins

Since XMH95 arrests the cell cycle and triggers apoptosis, the expression of components of the cell cycle machinery and members of the BCL2 family was examined (Fig. 2D). After 18 hours, a consistent and significant upregulation of *CDKN1A* expression was observed. This key member of the cyclin-dependent kinase inhibitors is regulated by various stressors and differentiation signals via both TP53-dependent and independent mechanisms [23–25]. In contrast, *CDKN2C*, another CDK that shows some alterations in LMS, was downregulated [26].

Analysis of apoptotic gene expression revealed that several BH3-only genes, such as *BBC3/PUMA, BIK*, *HRK* (Harakiri) and *PMAIP1/NOXA*, were upregulated after 18 hours of treatment (Fig. 2D). This upregulation of BH3-only genes could be responsible for the observed apoptosis induction. Therefore, we compared the expression of *BBC3/PUMA, BIK* and *PMAIP1/NOXA* in LMS SK-UT-1 cells and in immortalized HUtSMC. *PMAIP1/NOXA* was selected because it is abundantly expressed in SK-UT-1 cells, whereas *HRK* was discarded due to its very low abundance (Fig. S2B). The qRT-PCR analysis showed that all three genes are upregulated in SK-UT-1 cells but not in the non-tumorigenic HUtSMC cells, which are resistant to XMH95-induced apoptosis (Fig. 2E). Since *BIK* mRNA was not amplified in HUtSMC cells, we examined the RNA-seq data to understand the basal expression level. Normalized counts analysis showed that *BIK* is almost not expressed in HUtSMC cells (Fig. 2F).

Induction of apoptosis, caspase activation and BBC3/PUMA upregulation was confirmed by immunoblot analysis (Fig. 2G). We also analyzed the induction of DNA damage and particularly of double-strand DNA (dsDNA) breaks by XMH95. Phosphorylation of histone variant H2AX (γH2AX positivity) occurred later after treatment, in parallel with activation of Caspase-3. This can be explained as a consequence of caspase-dependent ICAD processing [27].

To prove the importance of BCL2 family members in the apoptotic response to XMH95, SK-UT-1 cells were engineered to overexpress BCL-XL/BCL2L1 (Fig. 2H). In the presence of elevated BCL-XL levels, XMH95 induced less cell death. Etoposide was used as a control (Fig. 2I). Next, we compared the dependence of XMH95 on BCL-XL with doxorubicin, a standard treatment for LMS. XMH95 5 µM and doxorubicin 0.1 µM induced a similar percentage of cell death in SK-UT-1 cells after 48 hours (79% ± 0.01 and 65% ± 0.07). The percentage of cell death reduction in cells expressing BCL-XL was similar after treatments with XMH95 or doxorubicin at 22.4 ± 4.9 and 19 ± 8.5, respectively.

We also confirmed that XMH95 does not induce dsDNA breaks by analyzing the occurrence of γH2AX positivity in HUtSMC and SK-UT-1 expressing BCL-XL treated with XMH95 for different time periods. Here, γH2AX was not increased after treatment (Fig. 2J). We can therefore assume that XMH95 does not induce dsDNA breaks.

### XMH95 triggers different transcriptomic adaptations in immortalized HUtSMC and SK-UT-1 LMS cells

**Fig. 3:**
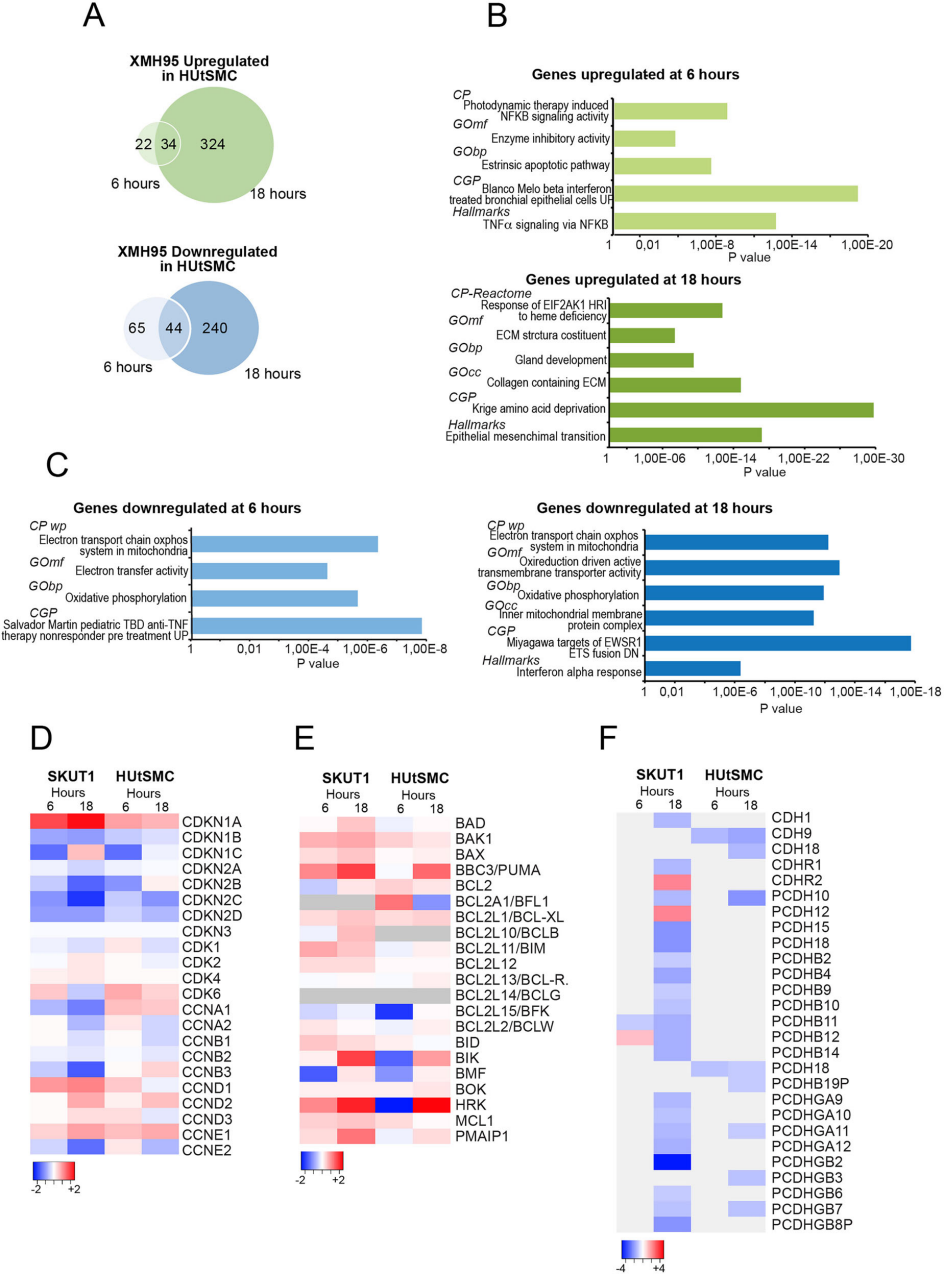
Specific transcriptional adaptations mark the differential apoptotic responses of non-transformed and cancer cells to XMH95. **A** Venn diagrams showing the number of transcripts upregulated in HUtSMC after 6 or 18 hours of treatment with 10 µM of XMH95. **B** Bar plots of functional enrichments using the GSEA and the Molecular Signatures Database (MSigDB) tools. The analysis was performed for genes upregulated after 6 and 18 hours of treatment with XMH95 in HUtSMC. Only the top terms are indicated. The detailed data are shown in Tables S6 and S7. **C** Bar plots of functional enrichments using the GSEA and the Molecular Signatures Database (MSigDB) tools. The analysis was performed for genes downregulated after 6 and 18 hours of treatment with XMH95 in HUtSMC. Only the top terms are indicated. The detailed data are shown in Tables S8 and S9. **D** Heatmap comparing the expression levels between HUtSMC and SK-UT-1 cells (log2 fold change relative to untreated cells) of genes encoding for the cell cycle machinery in response to XMH95 at 6 and 18 hours of treatment. **E** Heatmap comparing the expression levels between HUtSMC and SK-UT-1 cells (log2 fold change relative to untreated cells) of genes encoding BCL2 family members in response to XMH95 at 6 and 18 hours of treatment. **F** Heatmap comparing the expression levels between HUtSMC and SK-UT-1 cells (log2 fold change relative to untreated cells) of Cadherins and Protocadherins grouped in the GO-BP category “Homophilic cell adhesion” in response to XMH95 at 6 and 18 hours of treatment.

The differential effect of XMH95 on the survival of non-transformed smooth muscle cells and highly aggressive LMS cells, prompted us to compare the transcriptomic adaptations to XMH95 treatment in these two different cell models. Again, analyses were performed at 6 and 18 hours after treatment. PCA analysis revealed the high reproducibility of the three biological replicates and a profound difference between SK-UT-1 cells and HUtSMC in terms of gene expression (Fig. S2C). Moreover, SK-UT-1 cells modulate many more genes than HUtSMC in response to XMH95 treatment (Tables S2, S7). This is particularly true for the late upregulated genes (2.6-fold more in SK-UT-1 cells). Similar to SK-UT-1 cells, early response genes are more frequently downregulated, while late response genes are more frequently upregulated (Fig. 3A). However, these differences were less pronounced in HUtSMC compared to SK-UT-1 cells.

Venn analysis showed that some genes, either upregulated or downregulated, are in common between HUtSMC and SK-UT-1 cells. Indeed, 39% and 41% of the early and late upregulated genes in HUtSMC are identical to SK-UT-1 cells. Among the downregulated genes, 37% and 30% are in common with SK-UT-1 cells, for early and late upregulated genes, respectively (Fig. S3A, B and Table S7). The enrichment analysis revealed some similarities between the two cell lines. Upregulation of TNF-α signaling and ECM production were enriched among the upregulated genes in both HUtSMC and SK-UT-1 cells. In contrast, oxidative phosphorylation and the interferon-α response were found among the downregulated genes (Fig. 3B, C and Tables S8/S9/S10/S11).

Next, we compared the effect of XMH95 on cell cycle and apoptosis in HUtSMC and SK-UT-1 cells. For cell cycle genes, the effect on mRNA levels was comparable between the two cell lines. On average, the response to XMH95 was more pronounced in SK-UT-1 cells, which was clearly seen in the case of *CDKN1A* (Fig. 3D). In agreement with qRT-PCR analysis (Fig. 2E), the induction of BH3-only members (*BBC3/PUMA, BIK* and *PMAIP1*) in response to XMH95 was much less pronounced in HUtSMC compared to SK-UT-1 cells (Fig. 3E). It is noteworthy that the two cell lines differ in the expression of *BCL2A1* (HUtSMC) and *BCL2L10* (SK-UT-1). The differential effect of XMH95 on the expression of BH3-only genes in SK-UT-1 and HUtSMC could explain the specific apoptotic response in cancer cells.

To better understand the molecular basis of the differential response to XMH95, we focused our analysis on the set of genes that were specifically up or downregulated in SK-UT-1 cells compared to HUtSMC after 18 hours of treatment (Fig. S3A, B). Interestingly, the enrichment analysis (Table S12) revealed that the GO-BP category “Homophilic cell adhesion” had the highest score (p = 1.82E-10). The heatmap confirmed the specific downregulation of several cadherins and protocadherins in SKU-TU-1 cells, with very few repressed in common in both cell lines (*PCDH10, PCDHGA11* and *PCDHGB7*) (Fig. 3F). TPM analysis confirmed that modulation of protocadherin expression can influence the dynamics of cell adhesion, with the *PCDHB* group being strongly expressed especially in SK-UT-1 cells (Fig. S4). Thus, modulation of cell-cell adhesion could also influence cell survival in response to XMH95.

### XMH95 becomes fluorescent upon binding to DNA

**Fig. 4:**
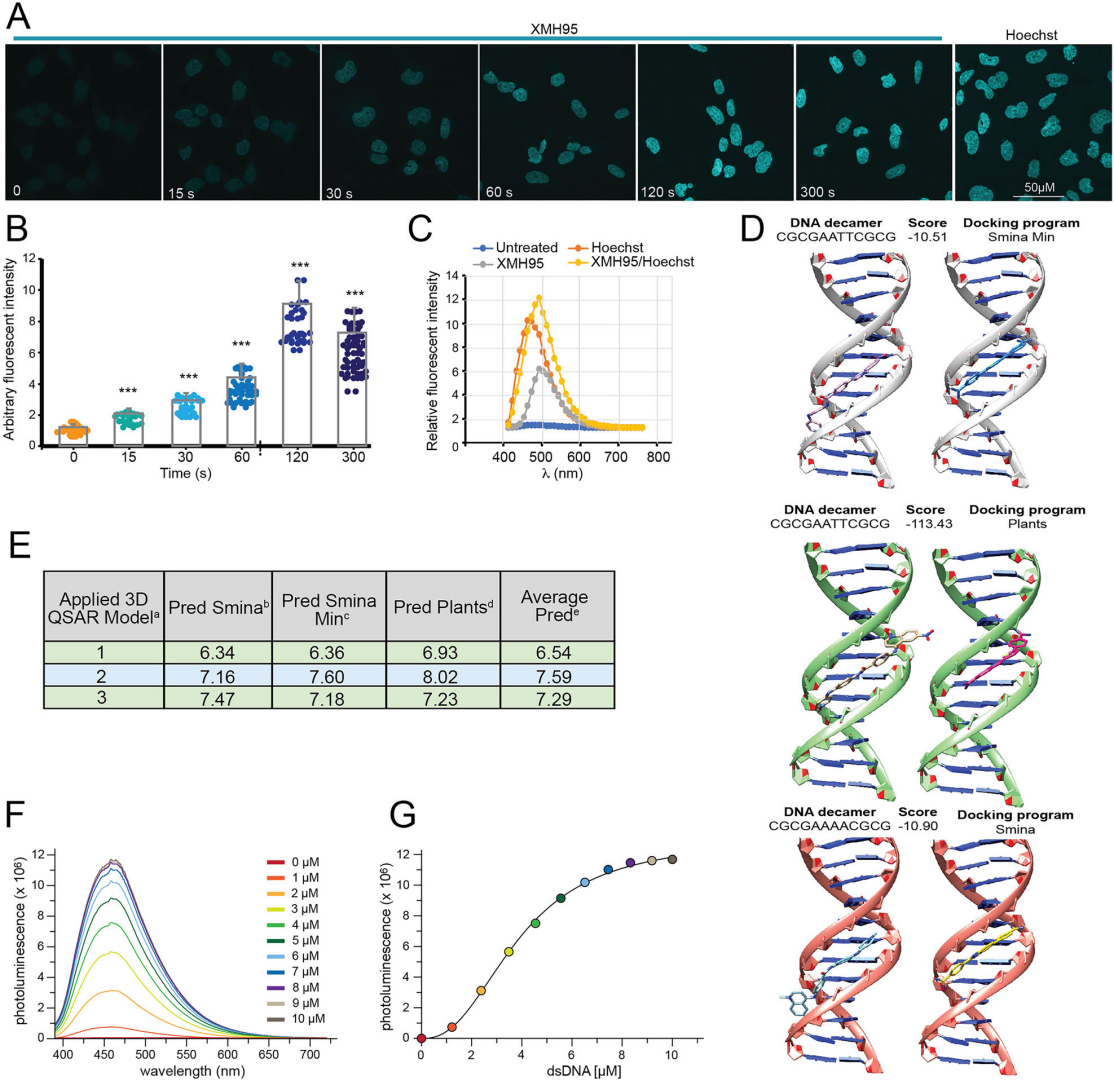
XMH95 emits fluorescence and binds the DNA in a similar way to Hoechst 33258. **A** SK-UT-1 cells were incubated with 10 µM of XMH95 for 6 hours. After fixation coverslips were observed by confocal microscopy. Photoactivation was performed on the confocal microscope stage immediately prior to image acquisition, using a 120-W mercury metal halide lamp (Leica EL6000 external light source, set to maximum intensity) combined with a 360/40 nm bandpass filter for the indicated seconds. Hoechst 33258 was used as control. **B** Quantification of fluorescence intensity in the cell nuclei from maximum intensity projections covering the whole cell volume. *p < 0.05, **p < 0.01, ***p < 0.001. **C** SK-UT-1 cells were treated with of XMH95 (10 µM) or Hoechst 33258 (10 µM) or both for 18 hours and then fixed. Emission lambda scans were recorded on a Leica TCS SP8 confocal microscope, with excitation at 405 nm and 10-nm detection bands from 415 nm to 775 nm. The mean fluorescence from the whole field is reported. **D** XMH95 (gold, cyan and pink colored aroms) predicted binding poses in three different dsDNA decamers conformations and compared with the co-crystallized ligands (magenta: DB1879, yellow: DB1476, blue: DB818). **E** Binding affinity prediction for XMH95 with the different 3D QSAR models using the docked conformations. (a: see Table S19; b: prediction with conformation predicted by Smina/Vinardo; c: prediction with conformation predicted by Smina/Vinardo and after complex minimization; d: prediction with conformation predicted by Plants/ChemPLP; e: average predicted p*K*_D_ values). **F** Photoluminescence titration spectra of XMH95 (10 µM) bound to different concentrations of dsDNA (0 - 10 µM) at 25 °C. **G** Titrations of XMH95 with dsDNA. The saturation limit is reached at a ratio of 1:1 XMH95/dsDNA, indicating a 1:1 stoichiometry.

Based on the structure of XMH95, we hypothesized that this compound could emit fluorescence. This property could allow us to evaluate its subcellular localization and help us understand its mechanism of action. We initially investigated the fluorescence properties of XMH95 in cultured cells. To this aim SK-UT-1 cells were incubated with XMH95 for 6 hours. After fixation, coverslips were mounted and observed under a confocal microscope. Hoechst 33258, a non-intercalating fluorescent dye that preferentially binds the minor groove of dsDNA to A-T-rich regions [28, 29], was used as a control. Upon initial inspection, the XMH95-treated cells did not appear to be stained. However, upon prolonged observation of the same field under UV light, we detected a fluorescent signal that was predominantly present in the nuclear compartment (Fig. 4A). To assess whether the fluorescence intensity of XMH95 within the cell varies over the time, we irradiated cells with UV light (120 W mercury metal halide lamp) for different time-lengths and performed confocal analysis. Fluorescence intensity and subcellular distribution of XMH95 is comparable to that of Hoechst (Fig. 4A). Quantitative analysis revealed a maximum fluorescence emission after 120 sec. of irradiation (Fig. 4B). Next, to identify the wavelength at which XMH95 emits at its maximum (λ_em,max_), we recorded emission lambda scans using a confocal microscope, with excitation at 405 nm and 10-nm detection bands from 415 nm to 775 nm. Again, we used Hoechst 33258 for comparison (Fig. 4C).

For XMH95 we observed a λ_em,max_ at 496 nm, while for Hoechst 33258 the measured λ_em,max_ was 465 nm, as previously reported [30]. Interestingly, a significant higher emission intensity was observed at 496 nm when the cells were co-incubated with both Hoechst 33258 and XMH95 altogether, as compared to XMH95 alone.

Based on these data and its chemical structure, we hypothesized that XMH95 might be capable to bind the dsDNA. To prove this, we initially performed an in silico analysis using a combination of 3D quantitative structure-activity relationship (3D QSAR) modeling and molecular docking. Our approach evaluated the binding affinities of XMH95 across multiple dsDNA decamers and used computational techniques to evaluate its structural and energetic properties. For the analysis, we selected 27 dsDNA decamer/ligand crystal complexes, available in the protein data bank (PDB) and associated with their relative dissociation constants (*K*_D_) values (see Materials and Methods). The Plants/ChemPLP and Smina/Vinardo docking program/scoring function pairs achieved a randomized docking accuracy higher than 50%, indicating that the binding conformation of a given docked molecule, whenever active, can be predicted with low root mean squared deviation (RMSD) in 50% of the cases (Table S20). Given the good docking performance, either Plants/ChemPLP or Smina/Vinardo pairs were used to dock the XMH95 compound into DNA decamers (Fig. 4D). Nevertheless, a very low correlation between the calculated p*K*_D_ and scoring functions (SF) scores was obtained, indicating that the SFs poorly predicted the molecular affinities, although the poses were in good agreement among the different docking simulations (Table S20). Hence, the SF values could not be used to evaluate the affinity of XMH95 for the dsDNA decamers. Joining molecular docking and 3D QSAR as an external SF, XMH95 was predicted to be a dsDNA ligand with average p*K*_D_ values in the range of 6.54-7.59 (Fig. 4E).

In summary, the computational analysis predicted: i) a binding affinity (p*K*_D_ = 6.93-8.02) of XMH95 to the dsDNA decamer using the docked conformation suggested by PLANTS; ii) that key residues within the dsDNA binding pocket interact with the carbamimidoyl and phenyl groups of XMH95, stabilizing the complex; iii) that hydrogen bonding and π-stacking interactions play crucial roles in the affinity of XMH95 for dsDNA, and finally iv) the docked conformation of XMH95 into different dsDNA decamers has an average *K*_D_ value ranging from 25 to 280 nM.

The in silico predicted ability of XMH95 to bind dsDNA prompted us to better investigate its binding properties experimentally. As a control, we again used Hoechst 33258 (Fig. S5B). Hoechst 33258 can be excited by UV or violet light sources (l_em,max_ = 352 nm) and emits a broad spectrum of blue light with λ_em,max_ = 454 nm. The frequently studied palindromic AT-rich dodecanucleotide sequence 5’-CGCAAATTTGCG-3’ was selected for this analysis [31, 32]. Similar to Hoechst 33258, the titration spectra of XMH95 showed an increase in photoluminescence signal upon binding to dsDNA (Fig. 4F/G and S5C/D). The photoluminescence intensity depends on the dsDNA concentration, with maximum intensity achieved at equimolar (1:1) concentrations of dsDNA and compound (10 µM), suggesting a 1:1 stoichiometry. Although Hoechst 33258 exhibited an overall higher photoluminescence intensity than XMH95, the latter showed negligible background photoluminescence in the absence of dsDNA, resulting in a higher signal-to-noise ratio ( ~ 14-fold for Hoechst versus ~410-fold for XMH95) (Fig. S5E). Remarkably, no significant changes in the chemical properties of XMH95 were observed when the compound was irradiated at 265 nm and 365 nm for 2 hours (Fig. S5F/G). Prolonged irradiation of XMH95 in complex with dsDNA (1:1 molar ratio) resulted in a drastic decrease in photoluminescence intensity (Fig. S5H).

In summary, XMH95 appears to bind the minor groove of dsDNA, become fluorescent only when associated to dsDNA, while when incubated with cells its fluorescence is strongly enhanced after UV irradiation.

### Mitochondrial genes are specifically repressed by XMH95

**Fig. 5:**
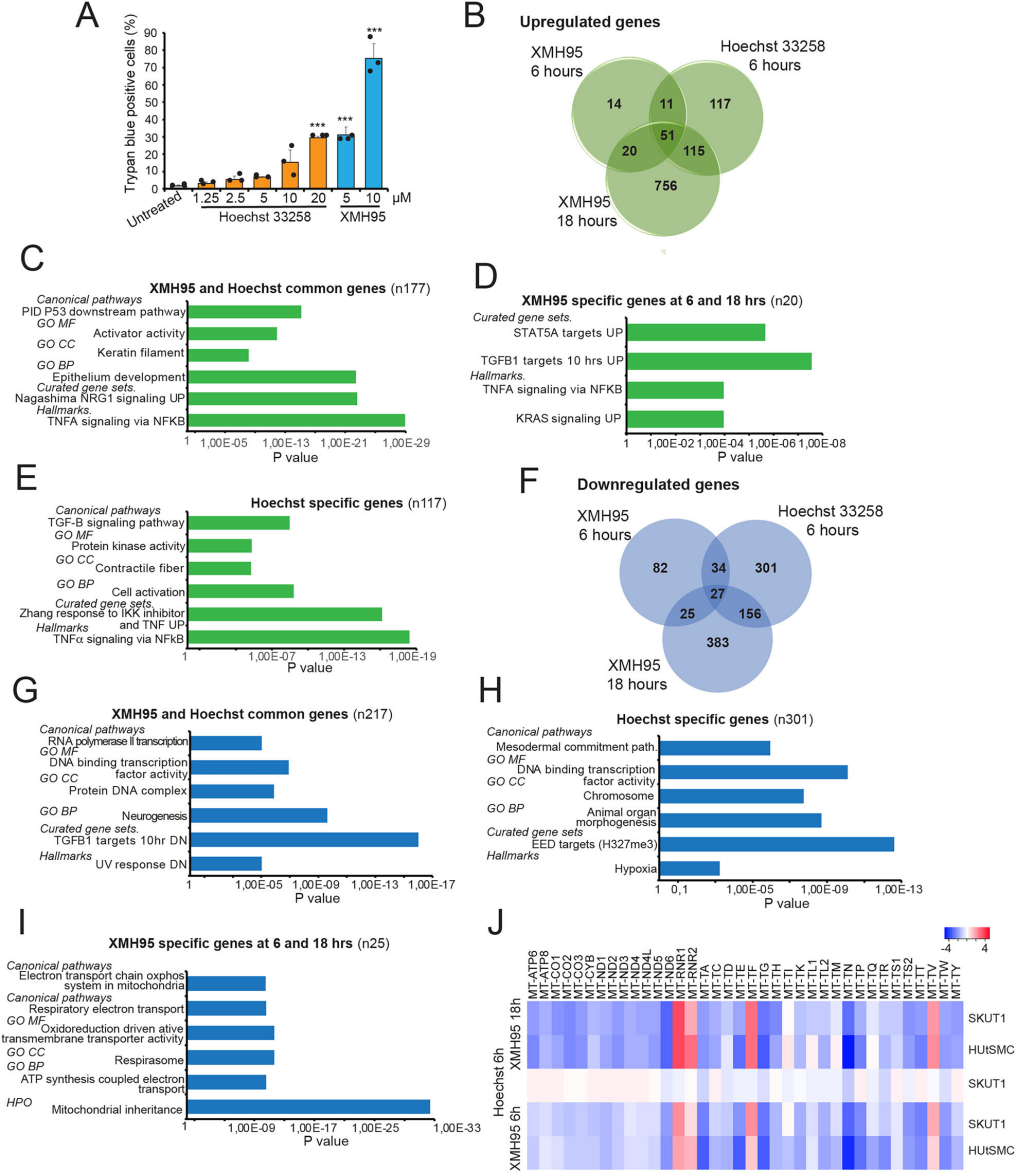
XMH95 differently from Hoechst 33258 triggers apoptosis and represses mitochondrial gene expression. **A** Cell death in SK-UT-1 cell line treated with the indicated concentrations of Hoechst 33258 or XMH95. Cell death was calculated 48 hours later as the percentage of cells positive to Trypan blue staining. Data are presented as mean + SD (n = 3). *p < 0.05, **p < 0.01, ***p < 0.001, relative to DMSO-treated cells (Untreated). **B** Venn diagram comparing the number of transcripts upregulated in SK-UT-1 cells after treatment for 6 or 18 hours with 10 µM of XMH95 or for 6 hours with 10 µM Hoechst 33258. **C** Bar plot of functional enrichments using the GSEA and the Molecular Signatures Database (MSigDB) tools. The analysis was performed for genes commonly upregulated by Hoechst 33258 and XMH95 in SK-UT-1 cells. Only the top terms are indicated. The detailed data are shown in Tables S10. **D** Bar plot of functional enrichments using the GSEA and the Molecular Signatures Database (MSigDB) tools. The analysis was performed for genes specifically upregulated by XMH95. Only the top terms are indicated. The detailed data are shown in Table S11. **E** Bar plot of functional enrichments using the GSEA and the Molecular Signatures Database (MSigDB) tools. The analysis was performed for genes specifically upregulated by Hoechst 33258. Only the top terms are indicated. The detailed data are shown in Table S12. **F** Venn diagram comparing the number of transcripts downregulated in SK-UT-1 cells after treatment for 6 or 18 hours with 10 µM of XMH95 or for 6 hours with 10 µM Hoechst 33258. **G** Bar plot of functional enrichments using the GSEA and the Molecular Signatures Database (MSigDB) tools. The analysis was performed for genes commonly downregulated by Hoechst 33258 and XMH95 in SK-UT-1 cells. Only the top terms are indicated. The detailed data are shown in Supplementary Table S13. **H** Bar plot of functional enrichments using the GSEA and the Molecular Signatures Database (MSigDB) tools. The analysis was performed for genes specifically downregulated by Hoechst 33258. Only the top terms are indicated. The detailed data are shown in Table S14. **I** Bar plot of functional enrichments using the GSEA and the Molecular Signatures Database (MSigDB) tools. The analysis was performed for genes specifically downregulated by XMH95. Only the top terms are indicated. The detailed data are shown in Table S15. **J** Regulation of mitochondrial gene expression in response to XMH95 or Hoechst treatments for the indicated times in HUtSMC and SK-UT-1 cells. Heatmap showing the expression levels (log2 fold change compared to untreated cells) of genes transcribed from the mitochondrial genome.

Since XMH95 can bind dsDNA similarly to Hoechst 33258, we wondered whether Hoechst 33258 could also induce apoptosis in SK-UT-1 cells. Compared to XMH95, Hoechst 33258 induced cell death much less potently, with only 30% cell death at a concentration of 20 μM (Fig. 5A). This finding is consistent with previous studies [33, 34]. To understand the molecular basis of this difference, we compared the transcriptomic profiles of SK-UT-1 cells treated with XMH95 or Hoechst 33258 for 6 hours. To determine whether differences in the timing of transcriptomic adaptations could be observed, we also included cells treated with XMH95 for 18 hours. After 6 hours, Hoechst showed a stronger transcriptomic effect compared to XMH95, with 294 genes upregulated versus 86 genes of XMH95 (Fig. 5B). Remarkably, 72% of the genes upregulated by XMH95 matched those of Hoechst 33258, suggesting a similar mechanism of action. Looking at the genes that were also upregulated after 18 hours of XMH95 treatment, 60% of the genes upregulated by Hoechst were also identical to those of XMH95 (Fig. 5B). The enrichment analysis was performed for three different groups of genes: genes common to XMH95 and Hoechst 33258, and genes specific to Hoechst 33258 or to XMH95. Elements of the TP53 response, TNF-α signaling and keratins are enriched among the frequently upregulated genes (Fig. 5C and Table S13). TNF-α response categories are also enriched among Hoechst-specific genes. Other categories include the TGF-β response and contractile fibres (Fig. 5E and Table S14). The XMH95-specific response shows similar enrichment for TNF-α and TGF-β signaling, but also specific enrichment for KRAS signaling (Fig. 5D and Table S15).

Next, we compared the downregulated genes. Similar to XMH95, Hoechst 33258 showed a stronger effect on gene repression (518 downregulated genes) than XMH95 (168 genes) after 6 hours (Fig. 5F). Analysis of fold changes in common genes confirmed the stronger effect of Hoechst 33258 on both activated and repressed genes (Fig. S6A/B). However, after 18 hours of treatment, XMH95 can affect the upregulation of common genes even more than Hoechst 33258. This observation suggests that the different intensities of modulation of common genes, which are mediated by the two compounds, may depend on their different stability, binding ratios, or kinetics of uptake. Transcription and transcription factors are the GO categories enriched among the common repressed genes by XMH95 and Hoechst 33258 (Fig. 5G and Table S16). Genes of TGF-β signaling and UV response are among the genes downregulated by the two compounds. Genes involved in chromosome organization and transcription, or hypoxia are specifically enriched in Hoechst-treated cells (Fig. 5H and Table S17). Notably, the 25 genes specifically repressed by XMH95 are enriched for mitochondrial respiration, electron transport and ATP synthesis (Fig. 5I and Table S18). Inspection of these 25 genes revealed that 72% of them are encoded by the mitochondrial DNA, confirming the enrichment of GO categories. Therefore, we analyzed the expression levels of all mitochondrially encoded genes in HUtSMC and SK-UT-1 cells treated with XMH95 or Hoechst 33258 (Fig. 5J). XMH95, but not Hoechst 33258, leads to downregulation of almost all mitochondrially encoded genes. Exceptions included the mitoribosomal RNA genes (*RNR1* and *RNR2*) and two mitochondrial tRNAs (mt-tRNA) *MT-TF* and *MT-TV* genes, which are instead upregulated. Importantly, *MT-TF* and *MT-TV* are adjacent to RNR2 and define its boundaries. Moreover, *MT-TV* acts either as a component of the mitoribosome or as a classical tRNA [35, 36].

In summary, our analysis showed that except for the cluster containing *MT-TF/RNR2/MT-TV/RNR1*, the suppression of mitochondrial gene expression is unique of XMH95 treatment with respect to Hoechst 33258, which otherwise shares several differentially regulated genes with XMH95.

### Repression of mitochondrial gene transcription is not sufficient to trigger cell death

**Fig. 6:**
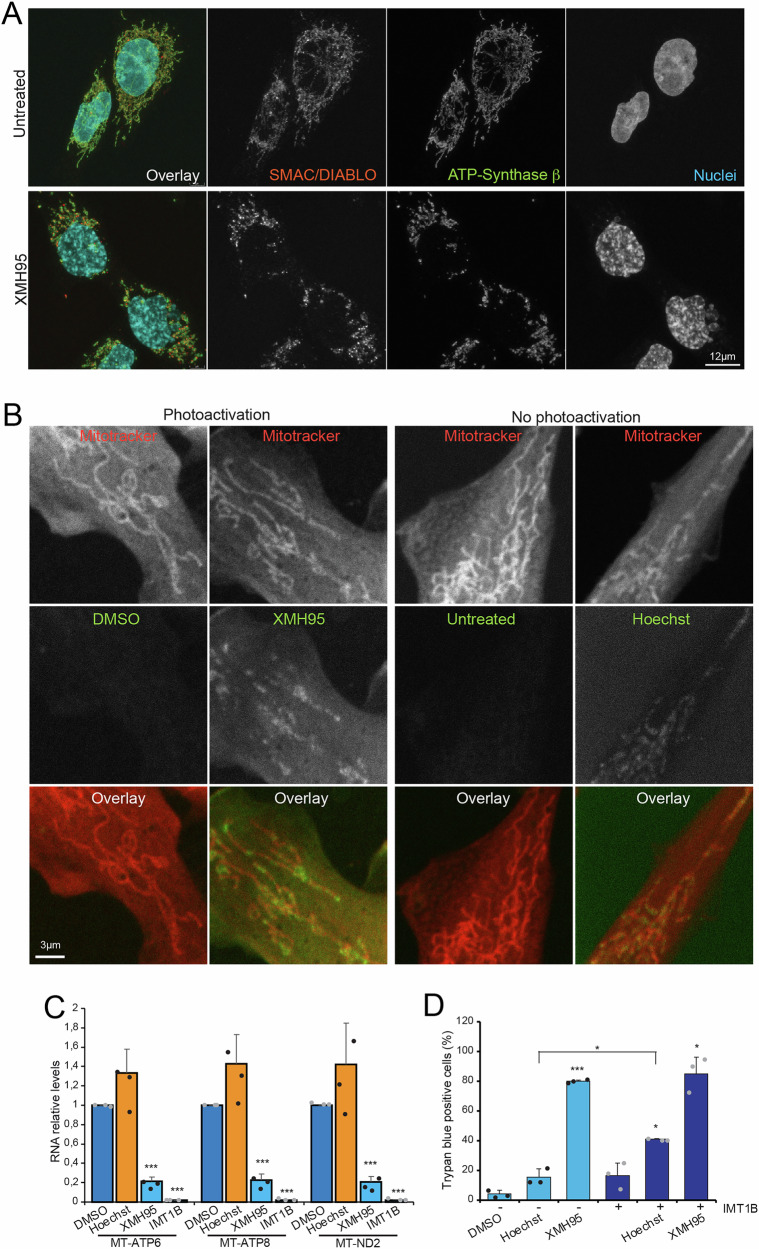
XMH95 binds the mitochondrial DNA, but repression of mitochondrial genes is not sufficient to induce apoptosis. **A** SK-UT-1 cells were treated or not with 10 µM of XMH95 for 18 hours. Immunofluorescence analysis was performed to visualize mitochondria morphology, using antibodies to visualize DIABLO/SMAC (red) and ATP synthase-β subunit (green). Nuclei were stained with Hoechst 33258 (cyano). Confocal images are shown in pseudocolors and were acquired with a Leica SP8 LSM. Bar 12 µm. **B** SK-UT-1 cells were grown in the presence of XMH95 (10 µM), Hoechst 33258 (10 µM) or DMSO for 4 hours. Cells were incubated in the presence of Mitotracker Red CMXRos (25 nm) for 1 hours before fixation. Photoactivation was performed on the confocal microscope stage immediately prior to image acquisition, using a 120-W mercury metal halide lamp (Leica EL6000 external light source, set to maximum intensity) combined with a 360/40 nm bandpass filter for 8 minutes. **C** Expression levels of the indicated mitochondrial encoded genes in SK-UT-1 cells treated with10 µM of XMH95, Hoechst 33258 or IMT1B. Incubation time was 18 hours for XMH95 and Hoechst 33258, and 48 hours for IMT1B. RNAs were extracted at the indicated processed for qRT-PCR. Data are relative to untreated cells. Mean ± SD; n = 3 or 4. *p < 0.05, **p < 0.01, ***p < 0.001, relative to DMSO-treated cells. **D** Cell death in the SK-UT-1 cells treated for 48 hours with 10 µM of XMH95, Hoechst 33258 or IMT1B. Cell death was calculated as the percentage of cells positive to Trypan blue staining. Data are presented as mean + SD (n = 3). p < 0.05, **p < 0.01, ***p < 0.001, relative to DMSO-treated cells.

After demonstrating a specific repressive effect of XMH95 on mitochondrial gene expression, we examined mitochondrial integrity after 18 hours of incubation with the compound (Fig. 6A). A time window before overt apoptosis onset. XMH95 triggers mitochondrial fragmentation, a common feature of cells entering apoptosis [37, 38]. However, in these mitochondria, the pro-apoptotic protein SMAC/DIABLO is still localized in the mitochondria, precluding the involvement of BAX and BAK and permeabilization of the outer mitochondrial membrane [38]. The localization of the ATP synthase-β subunit was used as a mitochondrial marker. In conclusion, suppression of mitochondrial gene expression cannot be associated with a fast activation of the intrinsic apoptotic pathway.

We hypothesized that XMH95 could suppress mitochondrial gene expression by binding to mitochondrial DNA and interfering with the transcription process. Figure 6B shows that XMH95 can stain discrete regions of mitochondria (visualized with Mitotracker) after UV irradiation. A similar pattern of mitochondrial staining, albeit much less bright, can be observed when cells were incubated with Hoechst 33258. Next, we validated by qRT-PCR the specific effect of XMH95 on the expression of mitochondrial genes (*MT-ATP6, MT-ATP8* and *MT-ND2*) (Fig. 6C). The non-competitive inhibitor of POLRMT, IMT1B, was used as a positive control for the repression of mitochondrial DNA transcription [39]. Only XMH95 and IMT1B were able to suppress mitochondrial gene expression. Hoechst 33258, on the other hand, was ineffective and confirmed the RNA-seq data.

Finally, we investigated whether the effect of XMH95 on mitochondrial genome expression was critical for the induction of apoptosis. Cells were treated with XMH95, Hoechst 33258 or DMSO in the presence or absence of IMT1B (Fig. 6D). Remarkably, IMT1B alone induced only a minimal percentage of cell death (14%). In addition, the combined treatment of Hoechst 33258 plus IMT1B showed an additive effect on cell death, which augmented from 26% (Hoechst 33258) alone to 41%. Although significant, the magnitude of this effect was much smaller compared to the effect observed with XMH95 alone (80%). In conclusion, suppression of mitochondrial gene expression alone is not sufficient to induce cell death, but it may contribute to the pro-apoptotic effect of XMH95 in highly proliferative LMS cells.

## Discussion

In this study, we have identified and characterized a novel compound, NSC-260594, named XMH95, which exhibits selective pro-apoptotic activity against highly aggressive LMS cells. This compound was previously identified as an RNA-binding and antiretroviral compound. It was shown to prevent the interaction of the HIV-1 Gag protein with (ψ)-stem-loop-3 RNA (SL3). This interaction is necessary for the encapsulation of the HIV-1 genome [40]. In another screening, NSC-260594/XMH95 was isolated as an antibacterial agent. Its activity has been linked to its ability to bind and inhibit *Escherichia coli* condensin MukBEF [41]. Recently, the NSC-260594/XMH95 was shown to be effective as an antineoplastic agent in a preclinical model of triple negative breast cancer (TNBC). NSC260594/XMH95 was suggested to inhibit the expression of MCL-1 (myeloid cell leukemia-1), a member of the BCL2 family with anti-apoptotic activity. This downregulation occurred primarily through the downregulation of WNT signaling proteins [42]. Although our study confirmed the key role of the mitochondrial apoptotic pathway in XMH95 activity, we did not observe the downregulation of *MCL-1* mRNA levels or enrichment of WNT signaling pathways in the RNA-seq experiments. Moreover, MCL1 can be proteolytized by caspases, so its downregulation is a common feature of the intrinsic apoptotic pathway and could also be triggered by additional mechanisms [43–45]. Instead, we observed upregulation of several BH3-only genes, possibly in response to a combination of nuclear and mitochondrial stress induced by XMH95. The upregulation of the BH3-only genes *PUMA/BBC3, PMAIP1/NOXA* and *BIK* was less pronounced in immortalised HUtSMC than in SK-UT-1 cells and could explain the very low toxicity of the compound in non-transformed cells. This different response is of interest from a therapeutic point of view.

We first identified XMH95 as a potential candidate for binding to a hydrophobic groove of the transcription factor MEF2D and thus blocking its interaction with multiple partners, including class IIa HDACs. Although we have not investigated whether XMH95 can bind the hydrophobic groove of MEF2, we have shown by indirect competitive assays that XMH95 cannot prevent the binding of an HDAC4-derived peptide to MEF2D. Therefore, XMH95 cannot be used as an inhibitor of the interaction between MEF2s and class IIa HDACs.

Further studies suggested instead that XMH95 can bind to dsDNA, specifically to the minor groove. Indeed, we showed that XMH95 can accumulate in vivo in cell nuclei and that in vitro it can bind the 5’-CGCAAATTTGCG-3’ sequence, a dodecamer commonly used to study the properties of minor groove dsDNA binders [29]. In addition, combination of 3D QSAR and docking approach provided a comprehensive understanding of the interaction of XMH95 with DNA. The specificity of the ligand for the minor groove of DNA can be attributed to favorable electrostatic and steric contacts. Binding of XMH95 to dsDNA appears to be mediated by hydrogen bonding and π-stacking interactions. By incorporating 3D-QSAR as an external scoring function, the reliability of the docking predictions was improved, highlighting their potential for DNA ligand modelling.

These computational studies resulted in a p*K*_D_ value for XMH95 that should be in the range of 6.54-7.59. However, further detailed biophysical studies are needed to accurately calculate the association and dissociation rate parameters and to better understand the optimal nucleotide sequence recognized by XMH95, as is the case for other minor groove binders [46].

In the past, several compounds have been identified and characterized as minor groove DNA binders. These compounds have been intensively studied as potential therapeutics or as specific DNA probes [46–49]. It is noteworthy that Trabectedin/Yondelis, a marine-derived natural product that binds the minor groove of dsDNA, among other effects, has shown efficacy in the treatment of leiomyosarcoma and liposarcoma compared to other soft tissue sarcomas [10, 50]. Trabectedin can inhibit transcription by i) blocking the elongation of RNApol II and promoting its degradation and ii) interfering with the binding of some TFs to their consensus sequences [51]. Trabectedin can also trigger an accumulation of DNA damage, which can be caused by various mechanisms [51–53]. In contrast, XMH95 is unable to induce double-strand DNA breaks. Consequently, gene expression studies in cells treated with XMH95 showed no signs of increased expression of genes involved in the DNA repair response.

In our comparative studies, we used Hoechst 33258, a well-known DNA minor groove binder. Compared to XMH95, Hoechst 33258 is a much less potent inducer of apoptosis in LMS cells. Comparative transcriptomic analyses between XMH95 and Hoechst 33258 showed that most of the changes in gene expression are superimposable between the two compounds. A result that further confirms the activity of XMH95 as a minor groove binder. The main difference between the two compounds was shown to be the repression of several mitochondrial genes, except for the mitoribosomal RNA genes and the two flanking tRNAs. Both DNA strands are involved in the transcription of the mitochondrial genome, and two long RNA filaments are produced, the light strand (LS) and the heavy strand (HS), which differ in nucleotide context. Their transcription is controlled by different promoters (LSP and HSP) [54–56]. The LSP controls the transcription of a long RNA that generates 8 tRNAs and the MT-ND6 gene. More recently, a second LS promoter has been identified [57].

Although two HSPs have been proposed in the past to control HS transcription to explain the much greater abundance of MT rRNAs compared to MT mRNAs, the different stability of the two types of RNA accounts for this difference [36, 54–57]. Therefore, we cannot exclude that XMH95 can repress all mitochondrial transcription. The downregulation of MT RNAs compared to MT rRNAs may be due to their intrinsic instability, which causes rapid decay once transcription is turned off [36, 58]. The ability of XMH95 to bind mitochondrial DNA underpins a possible direct effect on mitochondrial transcription. It is important to emphasize that the suppression of mitochondrial genes is a very early response to XMH95 treatment and not a consequence of mitochondrial damage due to the onset of the apoptotic response.

Although the effect on mitochondrial gene expression is important for the apoptotic response, it alone is not sufficient to kill LMS cells. In fact, the inhibitor of POLRMT, which strongly suppresses mitochondrial gene expression, does not trigger an efficient apoptotic response. Only the combination of Hoechst 33258 with the POLRMT inhibitor can induce significant cell death. Therefore, we hypothesize a synergistic nuclear and mitochondrial transcriptional stress as the main cause of XMH95-induced apoptosis in LMS cells.

Another interesting feature of XMH95 is its ability to develop fluorescence when it is in complex with dsDNA and irradiated with UV light. When cells are treated with XMH95, prolonged exposure to UV light significantly increases fluorescence emission. Although we have not yet elucidated the molecular basis of these phenomena, the ability of XMH95 to switch its optical properties opens the door to various applications aimed at studying the dynamics of nuclear structure and DNA organization. Since dsDNA enhances the fluorescence of XMH95, this could affect the definition of its subcellular localization, possibly limiting its visualization to DNA binding. Therefore, we cannot exclude the possibility that XMH95 also accumulates in other cellular compartments where its fluorescence cannot be stimulated.

Our study also has some limitations. First, we did not provide mechanistic details on the interaction of XMH95 with DNA and the possible effects of the compound on the structure of DNA. For this purpose, the crystal structure of XMH95 in complex with the dodecanucleotide used in the in vitro binding study is required. A second limitation concerns the molecular mechanisms underlying the conditional fluorescence properties of XMH95. Currently, the reasons for the differences in fluorescence switching and intensity observed when XMH95 is incubated with cells or purified DNA are unclear. The fixation procedure, the mounting medium and the presence of other macromolecules could in principle influence the properties of XMH95. Finally, we have not performed studies in animals, although they have been performed previously by another research group [42]. The main reason for this is the poor solubility of XMH95. In the future, it will be necessary to develop a specific delivery system, as we have already done with other compounds [59]. Alternatively, chemical modifications of XMH95 should be introduced with the aim of increasing its solubility. Of course, any modification should be thoroughly tested for the preservation of dsDNA-binding and pro-apoptotic activities.

In summary, our studies have identified a new dsDNA-binding ligand and characterized its biological action as an antineoplastic agent. XMH95 may represent a new tool for molecular and cell biology studies and is also a promising compound for potential therapeutic applications due to its ability to modulate gene expression.

## Materials and methods

### Reagents

Antibodies used in this study were from: Thermo Fisher Scientific; β-Actin Monoclonal Antibody (15G5A11/E2, Cat# MA1-140; RRID:AB_2536844); ATP Synthase-β Monoclonal Antibody (4.3E8.D10 Cat# MA1-930; RRID:AB_2227740); Goat anti-Rabbit IgG (H + L) Highly Cross-Adsorbed Secondary Antibody, Alexa Fluor™ 546 (Cat# A-11035; RRID:AB_2534093); Goat anti-Mouse IgG (H + L) Cross-Adsorbed Secondary Antibody, Alexa Fluor™ 488 (Cat# A-11001; RRID:AB_346865). Cell Signalling Technology; Caspase-3 (D3R6Y) Rabbit mAb (Cat# 14220; RRID:AB_2798429); Bcl-XL (54H6) Rabbit mAb, (Cat# 2764; RRID:AB_2228008); Puma Antibody, Rabbit polyclonal (Cat# 4976; RRID:AB_2064551); Phospho-Histone H2A.X (Ser139) (D7T2V) Mouse mAb (Cat# 80312; RRID:AB_2799949) Anti-SMAC [60]. Sigma-Aldrich; Anti-Mouse IgG (whole molecule)–Peroxidase antibody produced in goat (Cat# A4416; RRID:AB_258167); Anti-Rabbit IgG (whole molecule)–Peroxidase antibody produced in goat (Cat# A0545; RRID:AB_257896). LICORbio; IRDye® 800CW Goat anti-Mouse IgG Secondary Antibody (Cat# 926-32210; RRID:AB_621842); IRDye® 800CW Goat anti-Rabbit IgG Secondary Antibody (Cat# 926-32211; RRID:AB_621843).

Chemicals used in this study were from: NCI Chemotherapeutic Agents Repository (USA), NSC-80735, NSC-80731, NSC-202386, NSC-37553, NSC-67436, NSC-91529, NSC-128606, NSC-84100, NSC-127133, NSC-308835, NSC-177365, NSC-158413, NSC-320218, NSC-335979, NSC-186200, NSC-341196, NSC-5856. Enimine (Kyiv Ukraine) NSC-260594/XMH95 4-[(1-methyl-6-nitroquinolin-4-ylidene)amino]-N-[4-[(1-methylpyridin-4-ylidene)amino]phenyl]benzamide. NKL-54 (N-(2-aminophenyl)-N’-[3-(trifuoromethyl)phenyl]heptanediamide) [20]. Sigma-Aldrich, Hoechst 33258 (Phenol, 4-[5-(4-methyl-1-piperazinyl)[2,5’-bi-1H-benzimidazol]-2’-yl]-, trihydrochloride) (Cat# 14530; CAS# 23491-45-4), IMT1B (LDC203974) ((S)-1-((R)-2-((4-(2-chloro-4-fluorophenyl)-2-oxo-2H-chromen-7-yl)oxy)propanoyl)piperidine-3-carboxylic acid), MedChemExpress (Cat# HY-137067; CAS# 2304621-06-3).

### Cell culture

LMS cell lines of different malignancy grade; SK-UT-1 [20], SK-LMS-1 [20] and DMR [61] were used. Primary Human Uterine Smooth Muscle Cell line immortalized with hTERT were previously described [60]. All cells were maintained in culture at 37 °C with an atmosphere of 5% CO2 in Dulbecco modified Eagle medium (DMEM) (Sigma-Aldrich Cat# D6546) supplemented with 10% of fetal bovine serum (FBS) (Sigma-Aldrich Cat# F7524), L-glutamine (2 mM, Euroclone Cat# ECB3000D), penicillin (100 U/ml), streptomycin (10 mg/ml) solution (Euroclone Cat# ECB3001D), and amphotericin B (250 μg/mL, Thermo Fisher Scientific Cat# ECM0009D). Cells were periodically tested for mycoplasma presence by microscopy after Hoechst 33258 (Sigma) staining and by qPCR for 16S rRNA detection using the following oligos. 5’-GGCGAATGGGTGAGTAACACG -3’Myco detection FW and 5’-CGGATAACGCTTGCGACCTATG-3’ Myco detection RV (Eurofins Genomics). The cell doubling times for HUtSMC and SK-UT-1 cells were calculated using the following equation:

$${\rm{doubling}}\; {\rm{time}}=\frac{24\;{\rm{hours}}\; {\rm{x}}\; {\rm{ln}}(2)}{\mathrm{ln}\frac{{\rm{cells}}\; {\rm{at}}\;72\;{\rm{hours}}}{{\rm{cell}}\; {\rm{at}}\;48\;{\rm{hours}}}}$$

### Retroviral infections

SK-UT-1 cells expressing BCL-XL/BCL2L1 were generated by retroviral infection, as previously described [62]. Briefly, retroviral vectors pWZL-Hygro and pWZL-Hygro/BCL-XL were transfected into the packaging cell line LinX-E or Ampho phoenix, by the calcium phosphate method or polyethylenimine (Sigma-Aldrich #919012). 60 hours later, viral supernatants were collected, filtered, supplemented with 8 µg/ml Polybrene, and combined with fresh medium to infect SK-UT-1 cells.

### Protein expression and purification

The transcription factor MEF2D was expressed and purified as previously described [19]. In brief, synthetic gene encoding MEF2D (1–95 aa) was cloned into pETite expression vector (Lucigen, Middleton, WI, USA) and produced using *E. coli* T7 SHuffle cells (New England Biolabs, Ipswich, MA, USA) in terrific broth (TB) media. Expression of MEF2D was induced at OD_600_ = 0.8 by adding 1 mM isopropyl-β-D-1thiogalactopyranoside (IPTG). Induced cells were maintained at 28 °C overnight. Cell pellets were resuspended in lysis buffer (10 mM HEPES pH 7.7, 30 mM NaCl, 0.5 mM EDTA, 0.5 mM DTT) and processed by sonication (Sonic Dismembrator Model 300, Thermo Fisher Scientific, Dreieich, Germany). MEF2D protein was purified via ion exchange chromatography using 20 mL of HiPrep CM FF 16/10 column (Cytiva, Freiburg, Germany) connected to an ÄKTA pure 25 M system (Cytiva, Freiburg, Germany) and equilibrated with 10 mM HEPES, pH 7.7. Elution was achieved by applying a 0–2 M (NH_4_)_2_SO_4_ linear gradient. Eluted fractions were collected, pooled, concentrated by using 3 kDa MWCO Macrosep Advance Centrifugal Device (Pall Corporation, Portsmouth, United Kingdom) at 4000 g and 4 °C on a Heraeus Multifuge X1R centrifuge (Thermo Fisher Scientific, Dreieich, Germany) and further purified via hydrophobic chromatography using a HiPrep Butyl FF 16/10 column (Cytiva, Freiburg, Germany) equilibrated with 10 mM HEPES pH 7.7, 2 M (NH4)2SO4, 0.5 mM EDTA, 0.5 mM DTT. Elution was performed by applying a salt gradient (0–2 M). Purest fractions were collected, pooled and further concentrated by using 3 kDa MWCO Macrosep Advance Centrifugal Device (Pall Corporation, Portsmouth, United Kingdom) at 4000 g and 4 °C on a Heraeus Multifuge X1R centrifuge (Thermo Fisher Scientific, Dreieich, Germany). The protein aggregates were removed by size exclusion chromatography (SEC) using a HiLoad 26/600 Superdex 75 prep grade column (Cytiva, Freiburg, Germany) connected to an ÄKTA pure 25 M system (Cytiva, Freiburg, Germany) and equilibrated with 10 mM HEPES pH 7.7, 200 mM NaCl, 0.5 mM EDTA, 0.5 mM DTT, 10% v/v glycerol. The fractions containing dimeric MEF2D protein were pooled and further concentrated by using 3 kDa MWCO Vivaspin 500 centrifugal concentrators (Merck, Darmstadt, Germany) at 4000 g and 4 °C on a Heraeus Multifuge X1R centrifuge (Thermo Fisher Scientific, Dreieich, Germany) to a final protein concentration of 12 mg/mL. Protein concentration was determined using a BioPhotometer D30 UV spectrophotometer (Eppendorf, Hamburg, Germany). Purified MEF2D protein was flash frozen in liquid nitrogen and stored at −80 °C.

### Chemical synthesis of peptides

Peptides were chemically synthesized by standard Fmoc (9-fluorenylmethoxycarbonyl) solid-phase peptide synthesis (SPPS) using a ResPepSLi automated peptide synthesiser (Intavis Bioanalytical Instruments, Köln, Germany) as previously described [63]. Briefly, Fmoc groups were removed using a 20% v/v solution of piperidine in DMF (180 µL x 2). Amino acid coupling was carried out twice for each Fmoc-amino acid (7.5 eq., 0.5 M solution in DMF) using the PyBOP/NMM coupling system (5.5 eq. 0.4 M / 9 eq. 4 M in DMF). Fmoc groups were removed using a 20% v/v solution of piperidine in DMF. Final acetylation capping was performed using a 5% v/v solution of acetic anhydride in DMF. DCM washes (0.3 mL x 5) were performed at the end of synthetic process. NMP was used as cosolvent in the peptide synthesis. The final peptides were deprotected (side-chain protected groups) and cleaved from the resin using a TFA/thioanisole/H_2_O/anisole/ODT mixture (90/2.5/2.5/2.5/2.5% v/v) for 3 hours at room temperature. The resin was removed by filtration and the peptides were precipitated with cold diethyl ether (50 mL). The precipitated peptides were resuspended in diethyl ether (30 mL x 2) and centrifuged (3 times). Finally, the peptides were dissolved in H_2_O:ACN (1:1), freeze-dried and lyophilized on a LIO-5PDGT (5Pascal, Milan, Italy). Crude peptides were dissolved in 100% v/v DMSO and purified by preparative reversed-phase high performance liquid chromatography (RP-HPLC) using a C18 SymmetryPrep functionalized silica column (7 μm, 7.8 mm × 150 mm, Waters, Millford, MA, USA) connected to a Waters Delta Prep LC 4000 System equipped with a Waters 2489 dual λ absorbance detector, a Waters 600 pump and a PrepLC Controller (Waters, Millford, MA, USA). A flow rate of 4 mL/min and a linear gradient (10% to 50% in 15 min.) with a mobile phase composed of eluant A (99.9% v/v H_2_O, 0.1% v/v TFA) and eluant B (99.9% v/v ACN and 0.1% v/v TFA) was applied. The purified peptides were freeze-dried. The purity and molecular mass of the peptides was assessed by LC-ESI as described below (Fig. S7 and Table S21). Concentrations of peptides were determined using a BioPhotometer D30 UV spectrophotometer (Eppendorf, Hamburg, Germany).

### Mass spectrometric analysis

The molecular mass of each peptide was determined by electrospray ionization mass spectrometry (ESI–MS) performed on a single quadrupole liquid chromatograph InfinityLab LC/MSD mass spectrometer coupled to a 1260 Infinity II LC system (Agilent Technologies, Santa Clara, CA, USA). The system operated with the standard ESI source and in the positive ionization mode. Peptides were run at a flow rate of 1 mL/min with a linear gradient of solvent B over 15 min. (solvent A: 99.9% v/v H_2_O and 0.1% v/v formic acid; solvent B: 99.9% v/v ACN and 0.1% v/v formic acid). The reversed-phase HPLC column was a Nucleosil 100-5 C18 (5 μm, 125 mm × 4 mm; Macherey-Nagel, Dueren, Germany). Data were acquired, processed and analysed using the Agilent OpenLAB CDS (Agilent Technologies, Santa Clara, CA, USA) and MestReNova (Mestrelab Research, Santiago de Compostela, Spain).

### Cell death assay

Cells were seeded at a concentration of 0.8×10^5^ cells/mL and treated 24 hours later. DMSO was used in untreated cells as control. Cells were harvested, resuspended and then incubated 1:1 with trypan blue (TB Gibco Cat# 15250-061) 0.1% and incubated for 1 min. at RT. The cell positivity to TB was determined through Countess II FL automated cell counter (Invitrogen).

### DEVDase activity assay

For the quantification of caspase-3/7 activities cells were seeded at a concentration of 0.8 ×105 cells/mL and treated the next day with different concentrations of XMH95 for 36 hours. Caspase-3/7 (DEVDase) activity was evaluated with the Apo-ONE® Homogeneous Caspase-3⁄7 Assay (Promega Cat# G7792) following the manufacturer’s instructions. Fluorescence was measured with the PerkinElmer EnSpire Multimode plate reader.

### Cell protein lysate and immunoblot

After the treatment cells were washed with a PBS 1X solution (137 mM NaCl, 2.7 mM KCl, 10 mM Na_2_HPO_4_, 1.8 mM KH_2_PO_4_, pH 7.4) and scraped with Leammly sample buffer 2x (20% glycerol, 12% Tris 1 M pH 6.8, 4% SDS, 0.01% bromophenol blue) completed with Phenylmethane Sulfonyl Fluoride (PMSF, 1 mM) and β-mercaptoethanol (2%). Protein lysates were sonicated for 5 min (30 sec. on and 30 sec. off, x5) at 4 °C and boiled for 10 min. Denatured proteins were loaded in a polyacrylamide gel to perform the electrophoresis (SDS-PAGE). Proteins were transferred to a 0.2 μm-pore-sized nitrocellulose membrane through a wet transfer system. Membranes were incubated with 5% non-fat dry milk (Serva) in TBST 1X (20 mM Tris, 150 mM NaCl, 0.1% Tween 20, pH 7.6), or, for phosphorylated protein detection, with Bovine Serum Albumin (BSA HyClone Cat# SH30574.02) 5%, for 1 hours at RT. Primary antibody were incubated at 4 °C overnight. The following dilutions of primary antibodies were used: anti-b -actin (1:10’000), anti-Caspase-3 (1:1000), anti-PUMA (1:1000), anti-gH2AX (1:1000). Secondary antibodies were incubated 1 hours at RT. The following dilutions of secondary antibodies were used: anti-rabbit HRP-conjugated (1:3000), anti-mouse HRP-conjugated (1:5000), goat anti-Mouse IgG IRDye® 800CW-conjugated (1:10’000), goat anti-Rabbit IgG IRDye® 800CW-conjugated (1:10’000). Membranes were developed on a photographic film after ECL incubation (Pierce, Invitrogen) or by detecting the fluorescence using OdisseyCLx (LI-COR).

### Retro-Transcription quantitative PCR (RT-qPCR)

RNA was extracted with the Quick-RNA MagBead kit (Zymo Research Cat# R2132) following the manufacturer’s instructions. 1 μg of extracted RNA was used for retro-transcription quantitative PCR (RT-PCR). RT-qPCR reaction mix included: 1x First Strand Buffer (Invitrogen), dithiotreitol (DTT; 10 mM; Merck Cat# 43825), oligo dT (1.6 μM; Sigma-Aldrich), dNTPs Mix (0.4 mM; Thermo Fisher Scientific Cat# 18427013), RNase inhibitor (0.5 U/μl; Thermo Fisher Scientific Cat# N8080119), and the Moloney Murine Leukemia Virus reverse transcriptase enzyme (M-MLV; 8 U/μl; (Thermo Fisher Scientific Cat# 28025013). RT-qPCRs were performed using SYBR green (Roche Cat# KK4600). Data were analysed by comparative threshold cycle (ΔΔCt) using *GAPDH* as normalizer. Oligonucleotides used were from Eurofins Genomics:

5’-CCCTTCATTGACCTCAACTACATG-3’ GAPDH FV

5’- TGGGATTTCCATTGATGACAAGC-3’ GAPDH RV

5’-GCCAGAGGAGAAATGTCTGA-3’ BIK FV

5’-AGTGTGGTGAAACCGTCCAT-3’ BIK RV

5’-ACCTCAACGCACAGTACGAG-3’ PUMA/BBC3 FW

5’-TAAGGGCAGGAGTCCCATGA-3’ PUMA/BBC3 RV

5’-AAGTTTCTGCCGGAAGTTCA-3’ PMAIP1/NOXA FW

5’-GCAAGAACGCTCAACCGAG-3’ PMAIP1/NOXA RV

5’-ATGGCCATCCCCTTATGAGC-3’ MT-ATP6 FW

5’-TAAGGGGTGTAGGTGTGCCT-3’ MT-ATP6 RV

5’-ATACTACCGTATGGCCCACCA-3’ MT-ATP8 FW

5’-GGGCTTTGGTGAGGGAGGTA-3’ MT-ATP8 RV

5’-AGCACCACGACCCTACTACT-3’ MT-ND2 FW

5’-TGGTGGGGATGATGAGGCTA-3’ MT-ND2 RV

### Immunofluorescence

For immunostaining cells were seeded on 13 mm coverslips at a concentration of 0.9×10^5^ cells/mL and treated the next day with 10 µM XMH95, 10 µM Hoechst 33258, or DMSO for 4, 6 or 18 hours. For XMH95 mitochondrial localization cells were incubated 1 hours with Mitotracker Red CMXRos (25 nM) before fixation. Cells were fixed with 3% paraformaldehyde (Merck) for 20 min. at RT, quenched with 100 mM glycine for 5 min. If antibody were used also permeabilization with 0.5% Triton X-100 for 5 min. and blocking with 0.1% BSA (Merck) in PBS for 1 hours at RT were performed. Primary antibodies were incubated at RT overnight with 0.1% BSA in PBS, secondary antibodies were incubated for 30 min. at 37 °C. Coverslips were mounted in Mowiol (Merck). Images were acquired using a Leica TCS SP8 confocal microscope (Leica Microsystems). The following antibodies were used: anti-SMAC [60] (1:100), anti-ATP synthase-b (1:100, #MA1-930, Thermo Fisher Scientific), Alexa Fluor 546-conjugated goat anti-rabbit IgG (1:100, #A11035, Thermo Fisher Scientific), Alexa Fluor 488-conjugated goat anti-mouse IgG (1:100, #A11001, Thermo Fisher Scientific).

### Computational methods - ligand dataset and target DNA

To create a dataset of minor groove DNA ligands a survey revealed that out of 201 DNA decamer/ligand crystal complexes available from PDB, only 27 were reported to have a dissociation constant KD. A few complexes contained the same ligand (DB1476) but complexed with different DNA decamers. A few ligands (Hoechst 33258 and DB1476) were replicated in the same DNA decamer but in different binding conformations (Table S22). The 27 crystals were added of the hydrogens and energy minimized and aligned on the DNA backbone. The DNA and the ligands were then stored separately in “lock” and “key” files, to obtain all the ligands superimposed usable for the 3D QSAR and the molecular docking assessment.

### 3D-QSAR modeling

All the 3D QSAR models’ elaboration were performed with the Py-CoMFA module as available at www.3d-qsar.com. Three predictive models were developed. Model 1: the full dataset of 27 ligands as listed in Data S3 was used for training, achieving an internal predictive power (r²) of 0.99. Model 2: The dataset was split into training (80%) and test (20%) sets, with a q²ext of 0.89 and SDEPext of 0.14 to develop some predictive evaluation. Model 3: Leveraged all dataset molecules but used parameters derived from Model 2, showing acceptable predictive power (Table S19). All the model showed very good statistical coefficients with r^2^ and q^2^ values up to 0.99 and 0.65, respectively. In model 2 it was also assessed that the splitting into training and test sets endowed the dataset with predictive power (q^2^_ext_ = 0.89).

### Molecular docking

Smina and Plants molecular docking programs were used with all their scoring functions (SF). A redocking procedure with either experimental or random ligand conformation was applied to select the best program/SF pair. All calculations were performed using the www.3d-qsar.com portal.

### Integration of 3D-QSAR and molecular docking

The 3D QSAR model was integrated as an external scoring function to predict the pK_D_ of the XMH95 docked conformation. XMH95 was docked with Smina/Vinardo and Plants/ChemPLP pairs and the docked conformations were subjected to all 3D QSAR models described above.

### Confocal microscopy studies

Coverslips were fixed, quenched and mounted as written above. Images were acquired on a Leica TCS SP8 confocal microscope equipped with a HC PL APO CS2 100×/1.40 oil objective. Cell fluorescent studies were performed on the confocal microscope stage immediately prior to image acquisition, by exposure for 0, 15, 30, 60, 120, 300 sec., to a 120-W mercury metal halide lamp (Leica EL6000 external light source, set to maximum intensity) combined with a 360/40 nm bandpass filter. Z-series spanning the whole cell volume were acquired using a 405 nm diode laser (0.5% transmission), with detection range of 420-510 nm and a PMT gain of 900 V. Fluorescence from the cell nuclei was quantified from maximum intensity projections using Leica Application Suite X (LAS X) version 3.5.5. For mitochondrial localization, a photoactivation of 8 minutes was performed and 2/3 planes Z-stacks were acquired using the 405 nm diode laser at 60% transmission, after removing lambda/4 STED filter, and detection parameters as above.

### Emission spectrum

Coverslips were fixed, quenched and mounted as explained above. Emission lambda scans were recorded on a Leica TCS SP8 confocal microscope equipped with a HCX PL APO lambda blue 63×/1.40 oil objective, with excitation at 405 nm and 10-nm detection bands from 415 nm to 775 nm. The mean fluorescence from the whole field was quantified using Leica Application Suite X (LAS X) version 3.5.5.

### RNA-seq

Cells were seeded at a concentration of 1×10^5^ cells/mL and treated the next day with 10 µM XMH95 or 10 µM Hoechst 33258, for 6 or 18 hours. RNA was extracted with the Quick-RNA MagBead kit (Zymo Research) following the manufacturer’s instructions. DNaseI step was included. RNA integrity was verified through agarose gel. 4 μg of each RNA sample was used for the RNA-Seq analysis. RNA-Seq library preparation and sequencing were performed at Biodiversa s.r.l. (Treviso, Italy) following Illumina specifications. Quality control for raw sequencing data was performed with FastQC program (v 0.11.9) and MultiQC program. Raw reads were clipped with Trimmomatic software (v 0.39). Reads with an overall sequence mean Phred quality lower than 28 were discarded. The selected reads were mapped to the Human reference genome downloaded from Ensembl (version 107) using STAR (v 2.7.3a). Transcript assembly and quantification were done with StringTie (v 2.1.5). A Python script (prepDE.py) was used to extract all the read count information directly from the files generated in the last step. Differential expressed genes (DEGs) were identified using DESeq2 library (v 1.42.0). The thresholds applied were log2 fold change ( | log2 FC | ) >1 and FDR < 0.05. The functional analysis was performed with enricher function in the ClusterProfiler (v4.10.0) library against gene set collections (msigdb.v7.5.1.symbols.gmt) downloaded from Molecular Signatures Database. Enrichment results are considered statistically significant with a Benjamini–Hochberg FDR adjusted p-value < 0.05. Venn diagrams were created with Venny (v2.1). Heat maps were created with Heatmapper [64].

### Fluorescence polarization competition binding assay

Fluorescence polarization (F*P*) values were determined using the equations (i), where S is the fluorescence intensity of emitted light parallel to excitation, *P* is the fluorescence intensity of emitted light perpendicular to excitation, and *G* is the correction factor that correct for instrument bias.

i $${\rm{FP}}=1000\frac{(S-{GP})}{(S+{GP})}$$

The *G* factor was experimentally determined using the probe alone. Binding of MEF2D to pHDAC4 peptide and different chemical compounds was measured by incubating different concentrations of each ligand with 0.26 μM fluo-pHDAC4 and 20 μM of MEF2D. Unlabeled peptide pHDAC4 (3-fold dilutions ranging from 1 to 100 μM) was used as positive control. Each mixture (100 μL) containing 10 mM HEPES, 200 mM NaCl, 1 mM DTT, 1 mM EDTA, pH 7.7, 0.26 μM fluo-pHDAC4, 20 μM of protein and the small molecule compound of interest (3-fold dilutions ranging from 1 to 100 μM) was transferred into black 96-well microplates (Optiplate, PerkinElmer) and incubated at room temperature for 30 min. or 16 hours. Controls samples without proteins and without ligands were also prepared to estimate fluorescence of displaced and bound probe, respectively. Polarization signals were recorded at 25 °C using an EnVision Multlabel Plate Reader (PerkinElmer) as described above. Data were analysed using GraphPad Prism software.

### Photoluminescence spectroscopy

Compound XMH95 and Hoechst 33258 were used from 2 mM stock solutions of the prepared by dissolving the compounds in 100% v/v/ DMSO (Merck) and stored at 25 °C in the darkness. Single-stranded DNA (ssDNA) palindromic dodecanucleotide AT-rich sequence 5’-CGCAAATTTGCG-3’ (1 µmol) was purchased from Eurofins with HPLC degree purity and used without further purification. Double-stranded DNA (dsDNA) was obtained by resuspending the ssDNA in low EDTA buffer (10 mM Tris, 0.1 mM EDTA, pH 8.0) at 100 µM and further annealed by slow cooling overnight after heating in a water bath to 95 °C for 5 min. Steady-state photoluminescence measurements were carried out at room temperature using a Horiba Jobin Yvon (Kyoto, Japan) Fluorolog-3 spectrofluorometer, equipped with a continuous-wave xenon arc lamp coupled to a double Czerny–Turner monochromator as excitation source and a single grating monochromator coupled to a Hamamatsu (Shizuoka, Japan) R928 photomultiplier tube as detection system. Suitable longpass filters were placed before the acquisition system. Titrations were performed at 25 °C in a 1 cm. path length quartz cuvette by gradually adding aliquots of dsDNA (final concentration ranging from 0 to 10 µM for XMH95 and from 0 to 8 µM for Hoechst 33258) to a constant concentration of XMH95 (10 µM) or Hoechst 33258 (8 µM) previously diluted into deuterated water purchased from Sigma-Aldrich (Darmstadt, Germany). Controls samples without dsDNA were also prepared to estimate fluorescence of compound alone. The spectra were collected after allowing an equilibration time of 20 min. Spectra were monitored from 390 to 720 nm. Data were analysed using GraphPad Prism software.

### Photodecomposition studies

Absorption spectra were recorded at 25 °C in a 10 × 10 mm fluorescence quartz cuvette (Hellma GmbH, Mullheim, Germany) with a Yoke 6000Plus double-beam spectrophotometer (Fengxian, China) and with an OceanOptics (Ocean Insight, Orlando, FL, USA) HR4000CG UV-NIR detector, fiber-coupled to an OceanOptics CUV-ALL-UV cuvette holder equipped with collimators. The cuvette holder was fiber-coupled to an OceanOptics DH-2000-BAL deuterium-halogen lamp. The angle between the source and the detector was 180°. The experimental equipment was further coupled with OceanOptics LED light sources centered at 265 and 365 nm (λ_ex_) for photodecomposition studies. The angle between the LED sources and the detector was 90°. Mixtures were prepared to contain 10 µM for XMH95 alone or in the presence of 10 µM dsDNA in water.

### Quantification and statistical analysis

Data were analysed using GraphPad Prism 9 or Microsoft Excel. Results are presented as mean ± standard deviation (SD) from at least three independent replicates. Comparisons between 2 groups were performed with a 2-tailed Student’s t test. Comparison of multiple samples were performed by 1-way ANOVA. p-value less than 0.05 was considered significant and indicated as followed: *p < 0.05, **p < 0.01, ***p < 0.005.

## Supplementary information

Original Data

Supplementary figures and tables

Table S1

Table S2

Table S3

Table S4

Table S5

Table S6

Table S7

Table S8

Table S9

Table S10

Table S11

Table S12

Table S13

Table S14

Table S15

Table S16

Table S17

Table S18

## Data Availability

Reagents and materials generated in this study are available from the corresponding author with the compilation of the material transfer agreement. RNA-seq data have been deposited at GEO: accession number GSE288920. Original western blot images have been deposited at Mendeley at), “Immunoblots”, Mendeley Data, V1, 10.17632/8b4y4v2mr2.1 and are publicly available as of the date of publication.
