# Supplementary figures and images for: Identification of a novel minor-groove DNA binder that represses mitochondrial gene expression and induces apoptosis in highly aggressive leiomyosarcoma cells
Source: Cell Death Discov. 2025 Nov 10;11:524. doi: 10.1038/s41420-025-02803-3 (PMC12603272; doi:10.1038/s41420-025-02803-3)

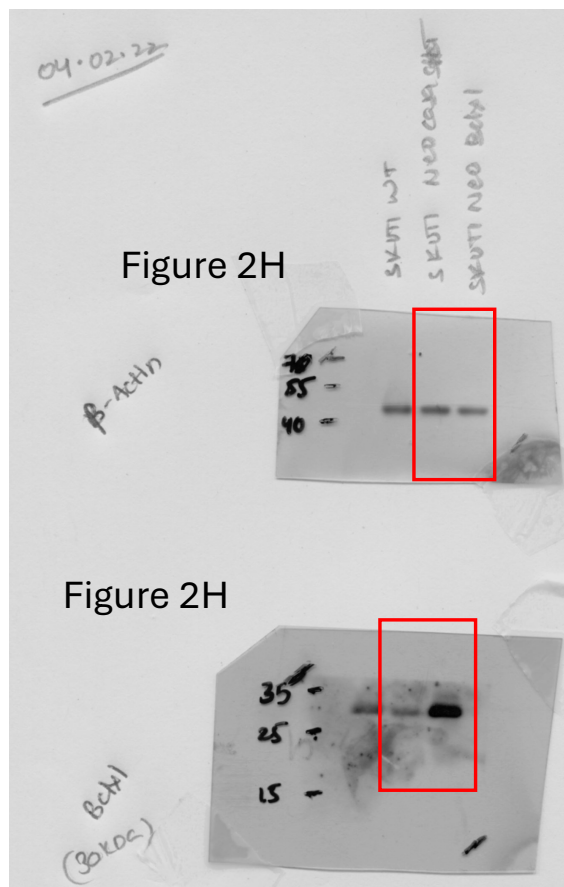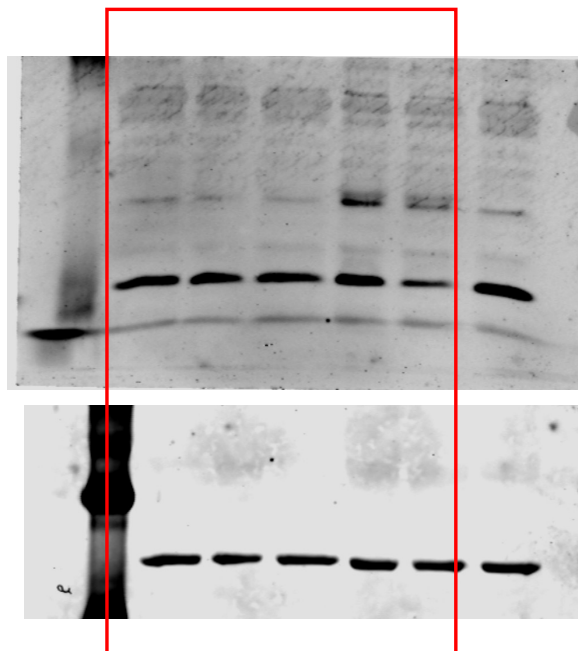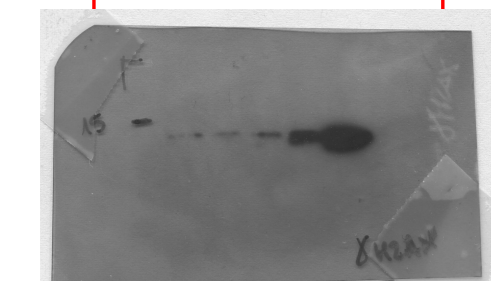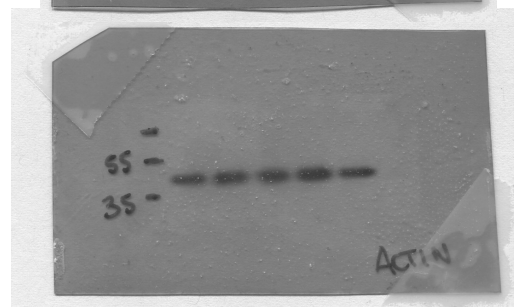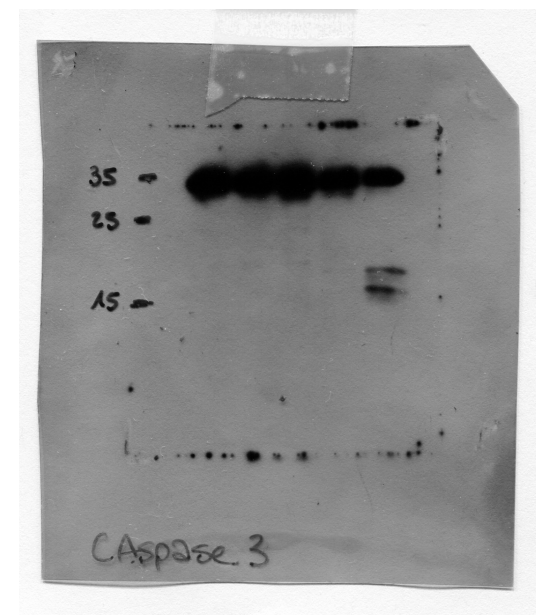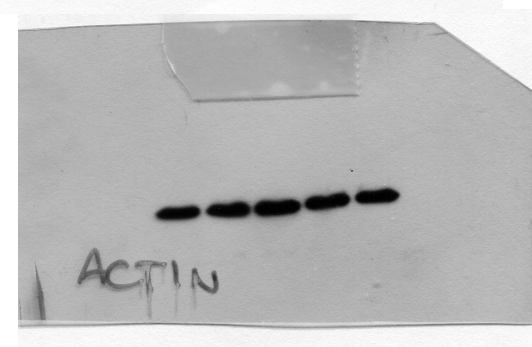

Figure 2G

Figure 2G

Figure 2G

Figure 2G

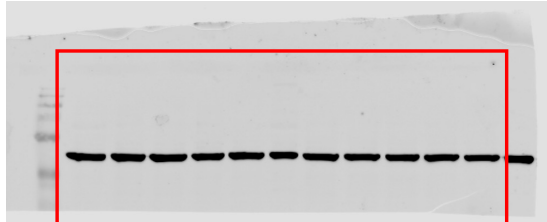

Figure 2J

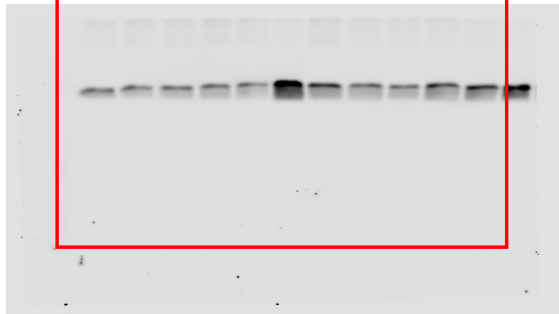

Figure 2J

Supplement: Supplementary file 1 — Original Data [file 41420_2025_2803_MOESM1_ESM.pdf]
