# Supplementary material for: Identification of a novel minor-groove DNA binder that represses mitochondrial gene expression and induces apoptosis in highly aggressive leiomyosarcoma cells
Source: Cell Death Discov. 2025 Nov 10;11:524. doi: 10.1038/s41420-025-02803-3 (PMC12603272; doi:10.1038/s41420-025-02803-3)
Supplement: Supplementary file 2 — Supplementary figures and tables [file 41420_2025_2803_MOESM2_ESM.pdf]

Supplemental information

Identification of a novel minor-groove DNA binder that represses mitochondrial genes expression and induces apoptosis in highly aggressive leiomyosarcoma cells

Supplementary figures

A

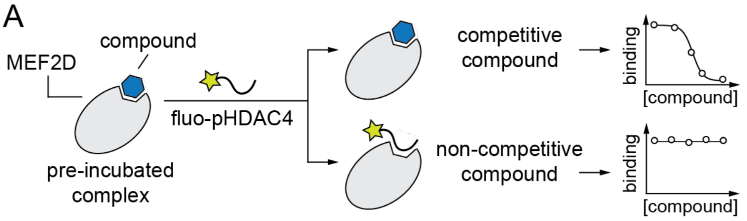

C

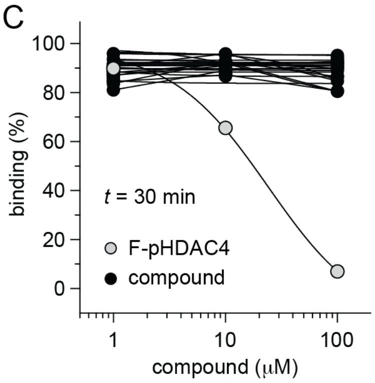

D

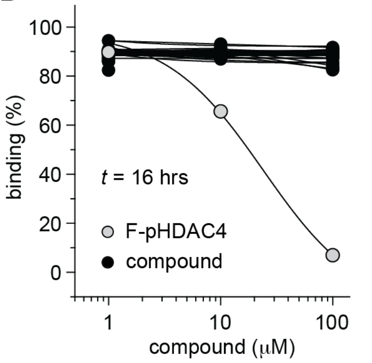

B

compound ID	30 min assay				16 hrs assay			
	0 μM	1 μM	10 μM	100 μM	0 μM	1 μM	10 μM	100 μM
pHDAC4	100	90±3	62±3	7±3	100	90±3	64±3	6±4
CMP5856	100	92±1	90±4	94±5	100	87±2	90±1	88±1
CMP37553	100	86±4	88±5	89±6	100	89±3	90±1	88±1
CMP67436	100	91±1	92±4	94±1	100	88±1	89±1	89±2
CMP80731	100	88±2	87±3	92±2	100	82±4	88±1	88±1
CMP80735	100	89±1	87±1	87±1	100	86±3	89±1	86±1
CMP84100	100	88±9	89±6	90±8	100	87±1	89±1	88±1
CMP91529	100	85±2	87±1	93±6	100	89±2	87±1	88±1
CMP127133	100	86±6	90±1	92±9	100	86±1	89±1	89±1
CMP128606	100	91±1	88±3	90±1	100	89±1	88±5	91±4
CMP158413	100	94±1	96±1	85±9	100	89±1	88±1	83±1
CMP177365	100	96±2	96±5	95±5	100	90±1	90±1	87±1
CMP186200	100	91±1	96±4	85±8	100	90±1	90±1	91±2
CMP202386	100	87±9	91±3	93±4	100	90±1	90±3	90±1
CMP260594	100	88±2	92±3	92±1	100	89±1	89±1	91±1
CMP308835	100	84±3	93±2	86±2	100	91±1	91±1	84±2
CMP320218	100	87±3	92±2	90±1	100	94±4	93±2	92±2
CMP335979	100	81±4	89±8	91±3	100	90±1	91±1	89±1
CMP341196	100	86±1	87±2	80±9	100	91±1	90±1	83±1

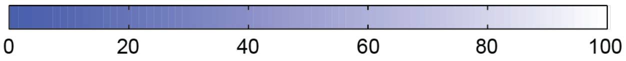

Figure S1. In vitro competition binding assay for detection of disruptors of MEF2D-HDAC4 interaction.

A. Schematic representation of the fluorescence polarization-based competition binding assay applied. The compounds and MEF2D-dsDNA complex are pre-incubated to form a tertiary complex before the addition of the fluorescent peptide probe fluo-pHDAC4;

B. Heat map displaying the results of the fluorescence polarization-based competition binding assay after 30 min. (left) or 16 h. (right) incubation with compounds. The extent of competition is indicated with color, ranging from blue (100% competition) to white (0% competition). Three concentrations (1, 10 and 100 μM) of un-labelled antagonist pHDAC4 peptide and small molecule compounds were used. Titration binding curves obtained by applying a fluorescence polarization-based competition assay in the presence of MEF2D-dsDNA complex, fluo-pHDAC4 probe and un-labelled antagonist pHDAC4 peptide and small molecule compounds after 30 min. C. and 16 h. (D). The mean fluorescence polarization values of at least three independent experiments are reported and plotted as function of MEF2D concentration. Data are presented as mean (dots) ± s.e.m. (bars).

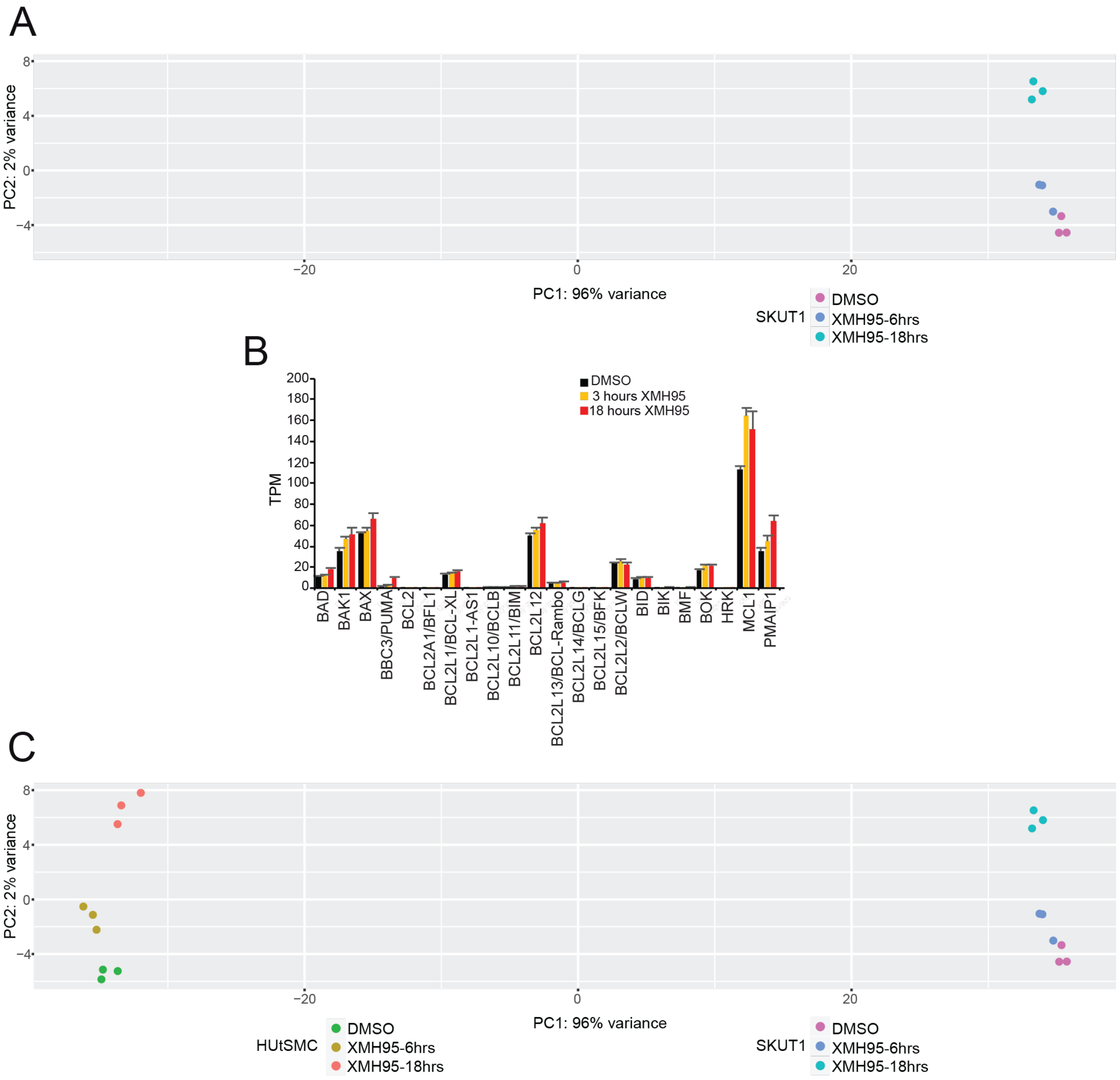

Figure S2. Transcriptomic studies in XMH95-treated cells.

A. PCA analysis of expression profiles of SK-UT-1 cells treated with 10 μM XMH95 for indicated times.

B. TPM values for BCL2 family members in SK-UT-1 cell and after treatments with 10 μM XMH95 for the indicated times. TPM was calculated from a gene model where isoforms were collapsed into a single gene.

C. PCA analysis of expression profiles of HUtSMC and SK-UT-1 cells treated with 10 μM XMH95 for indicated times.

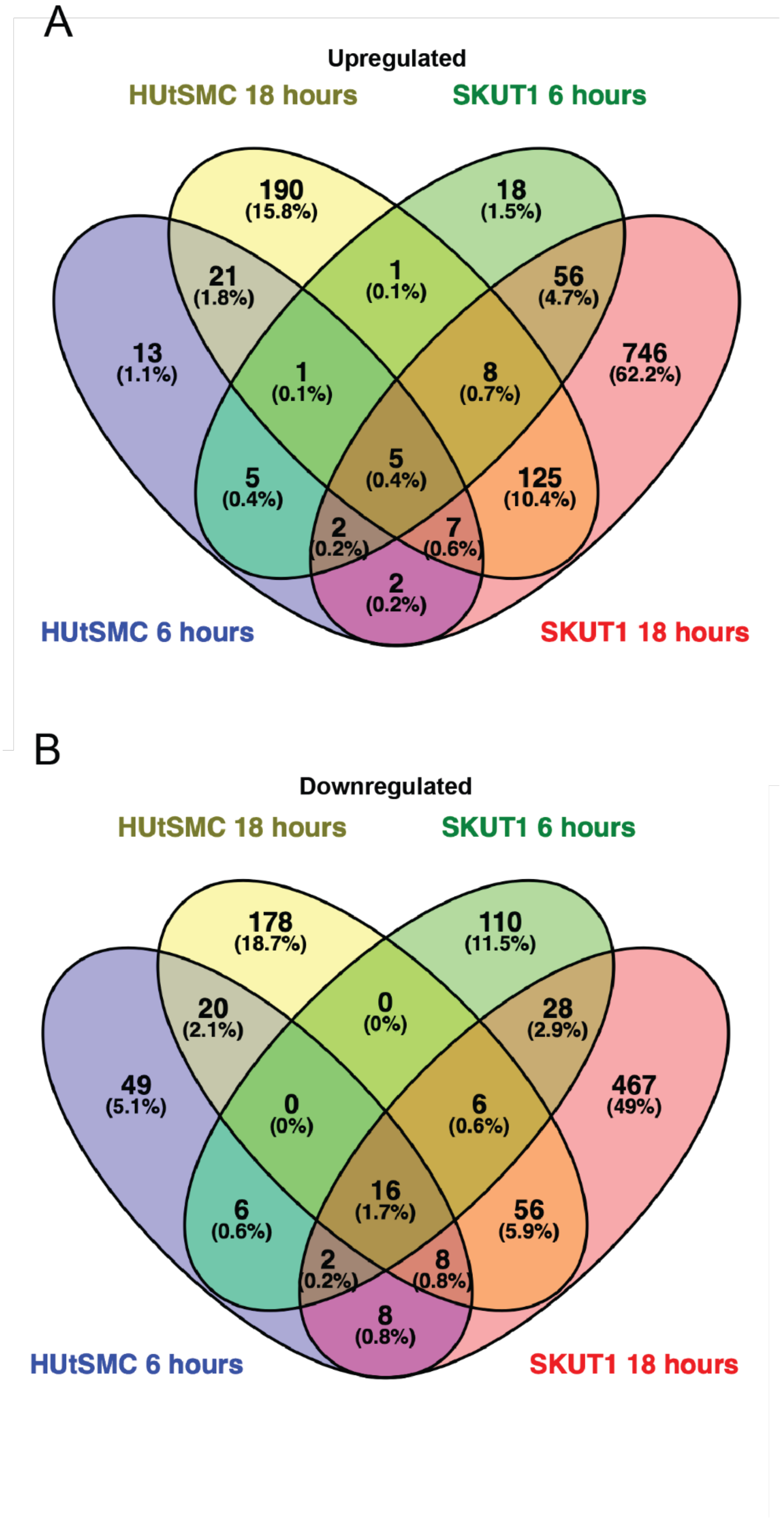

Figure S3. Differential gene expression in XMH95-treated cells.

A. Venn diagrams showing the number of upregulated and shared transcripts between SK-UT-1 and HUtSMC cells after 6 and 18 h. of treatment with 10 µM of XMH95.

B. Venn diagrams showing the number of downregulated and shared transcripts between SK-UT-1 and HUtSMC cells after 6 and 18 h. of treatment with 10 µM XMH95.

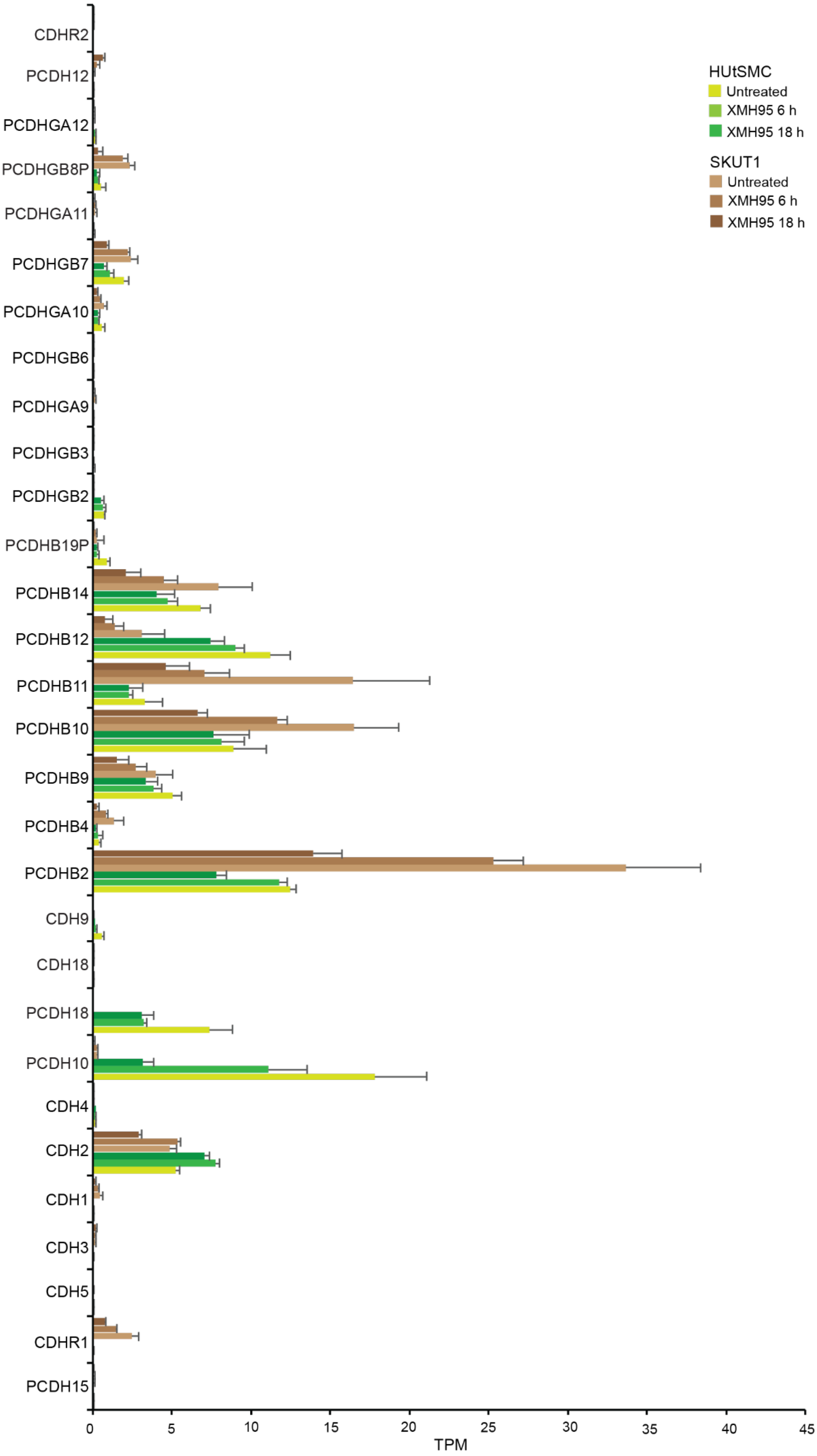
CDHR2
PCDH12
PCDHGA12
PCDHGB8P
PCDHGA11
PCDHGB7
PCDHGA10
PCDHGB6
PCDHGA9
PCDHGB3
PCDHGB2
PCDHB19P
PCDHB14
PCDHB12
PCDHB11
PCDHB10
PCDHB9
PCDHB4
PCDHB2
CDH9
CDH18
PCDH18
PCDH10
CDH4
CDH2
CDH1
CDH3
CDH5
CDHR1
PCDH15
0
5
10
15
20
25
30
35
40
45
TPM
HUtSMC
Untreated
XMH95 6 h
XMH95 18 h
SKUT1
Untreated
XMH95 6 h
XMH95 18 h

Figure S4. Cadherins and protocadherins expression in SK-UT-1 cells and HUtSMC after treatment with XMH95.

TPM were calculated from a gene model where isoforms were collapsed into a single gene. Cells were treated with 10 μM XMH95 for the indicated times

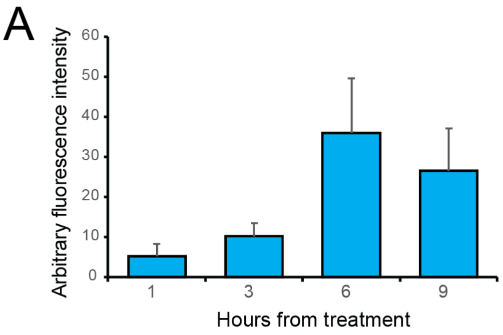

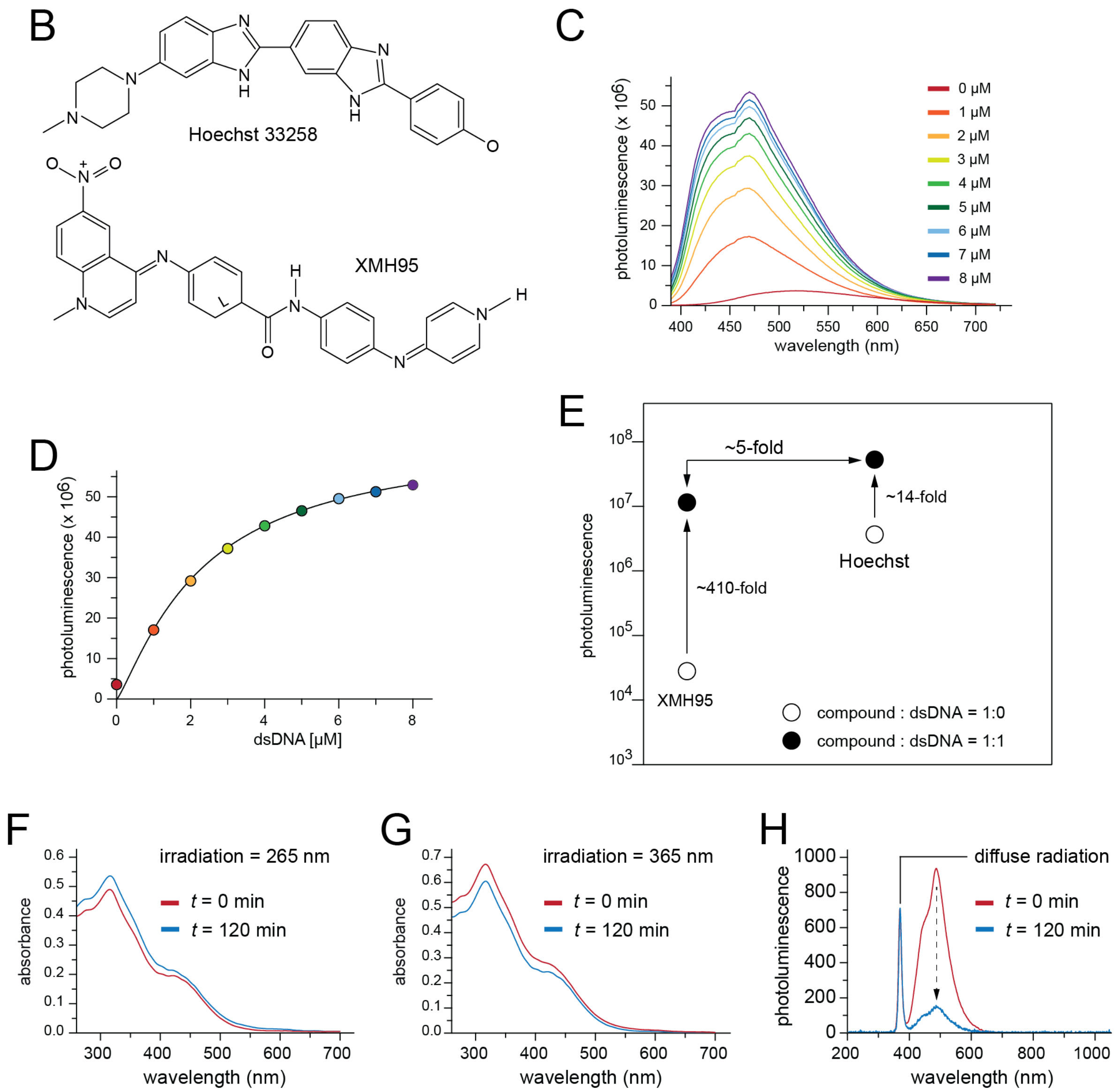

Figure S5. XMH95 binds the DNA.

A. Quantification of fluorescence intensity in the cell nuclei from maximum intensity projections covering the whole cell volume. Cells were incubated for the indicated time with XMH95 (10μM) before fixation. A minimum of 36 nuclei were counted for each time-point. S.D. indicates the variation of fluorescence intensity among nuclei.
B. Chemical structure of Hoechst 33258 and XMH95.
C: Photoluminescence titration spectra of Hoechst 33258 (8 μM) bound to varying concentrations of dsDNA (0 – 8 μM) at 25°C.
D. Titrations of Hoechst 33258 (8 μM) with dsDNA (0 - 8 μM).
E. Dot plot of the photoluminescence intensity measured for XMH95 (left) and Hoechst 34580 (right) in absence (white dot) or in presence of dsDNA (black dot).
F. Absorption spectra of XMH95 (10 μM) excited at 265 nm in water at 25°C. Spectra were monitored from 280 to 700 nm both at the start of the experiment (t = 0 min, red line) and after two hours of irradiation (t = 120 min, blue line);
G. As in (E) but XMH95 was excited at 365 nm.
H. Photoluminescence spectra of XMH95 (10 μM) bound to dsDNA (10 μM) at the start of the experiment (t = 0 min, red line) and after two hours of irradiation (t = 120 min, blue line) at 25°C. Spectra were monitored from 200 to 1000 nm.

A

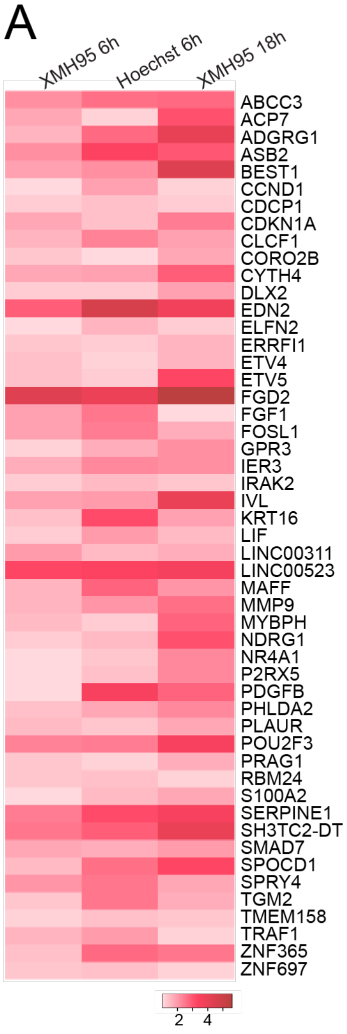

B

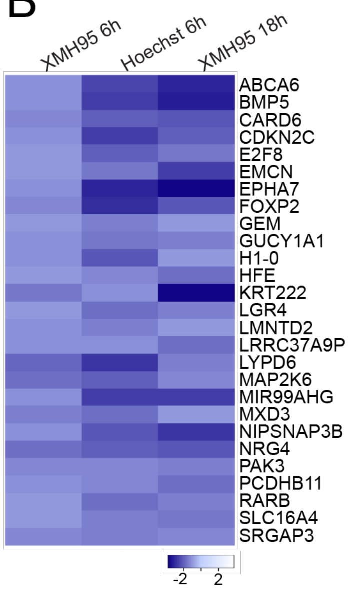

Figure S6. Fold changes of common Hoechst and XMH95 regulated genes.

A. Log2 fold change relative to untreated cells of commonly upregulated genes in response to Hoechst or XMH95 treatment. Treatment hours are indicated.

B. Log2 fold change relative to untreated cells of commonly downregulated genes in response to Hoechst or XMH95 treatment. Treatment hours are indicated.

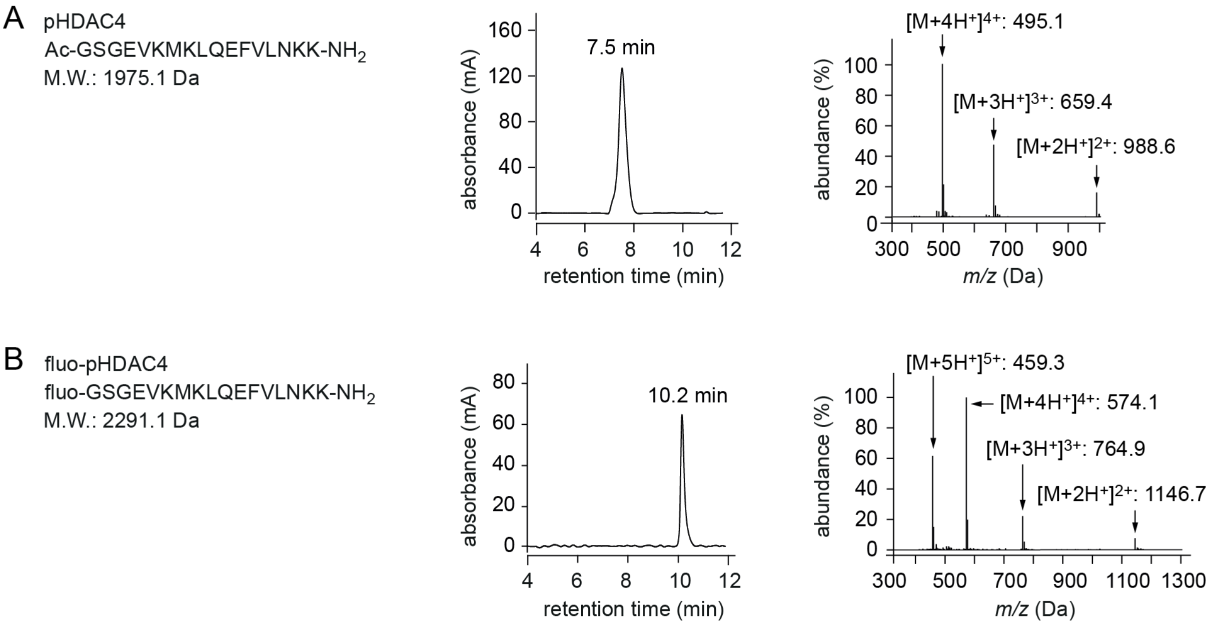

Figure S7. Characterization of the purified pHDAC4 peptides.
HPLC (left) and MS (right) analysis of purified peptides pHDAC4 (A) and fluo-pHDAC4 (B). Peptide stock solutions were injected into an Agilent 1260 Infinity HPLC system equipped with a Nucleosil 100-5 C18 Macherey-Nagel (5 mm, 125 mm x 4 mm) analytical column, and run at a linear gradient with a mobile phase composed of eluent A (99.9 % v/v H_2O, 0.1% v/v HCOOH) and eluent B (99.9% v/v acetonitrile and 0.1% v/v HCOOH) from 10% to 50% over 15 minutes at a flow rate of 1 ml/min. The absorbance at a wavelength of 220 nm is presented with corresponding retention time. The molecular mass of each peptide was determined by electrospray ionisation mass spectrometry (ESI–MS) performed on a single quadrupole coupled to a 1260 Infinity II LC system. The measured molecular weight of each peptide corresponds to expected mass.

Supplementary Tables

Table S1. Characteristics and structures of the 18 selected compounds

Table S2. Up and downregulated genes after XMH95 treatment in SK-UT-1 cells

Table S3. Functional enrichment of gene categories for genes upregulated after 6 h. of XMH95 treatment.

Table S4. Functional enrichment of gene categories for genes upregulated after 18 h. of XMH95 treatment.

Table S5. Functional enrichment of gene categories for genes downregulated after 6 h. of XMH95 treatment.

Table S6. Functional enrichment of gene categories for genes downregulated after 18 h. of XMH95 treatment.

Table S7. Up and downregulated genes after XMH95 treatment in HUtSMC cells

Table S8. Functional enrichment of gene categories for genes upregulated after 6 h. of XMH95 treatment.

Table S9. Functional enrichment of gene categories for genes upregulated after 18 h. of XMH95 treatment.

Table S10. Functional enrichment of gene categories for genes downregulated after 6 h. of XMH95 treatment.

Table S11. Functional enrichment of gene categories for genes downregulated after 18 h. of XMH95 treatment.

Table S12. GOBP enriched categories among genes specifically downregulated in SK-UT-1 cells.

Table S13. Functional enrichment of gene categories for genes upregulated by both XMH95 and Hoechst.

Table S14. Functional enrichment of gene categories for genes upregulated specifically in response to Hoechst treatment.

Table S15. Functional enrichment of gene categories for genes upregulated specifically in response to XMH95 treatment.

Table S16. Functional enrichment of gene categories for genes downregulated by both XMH95 and Hoechst.

Table S17. Functional enrichment of gene categories for genes downregulated specifically in response to Hoechst treatment.

Table S18. Functional enrichment of gene categories for genes downregulated specifically in response to XMH95 treatment.

Table S19. Results from 3D-QSAR modeling

#[a]	Model Info	TrSet[b]	TsSet[c]	PC[d]	r^{2e}	SDEC[f]	q^{2g}	$SDEP_{CV}^{h}$	$q^2_{ext}{}^{i}$	$SDEP_{ext}{}^{j}$
1	Model Derived from All Dataset Mols as Training Set	27		7	0.99	0.10	0.65	0.27	-	-
2	Model Derived from Dataset Split into Training and Test Sets (80:20)	21	6	5	0.98	0.11	0.37	0.37	0.89	0.14
3	Model Derived from All Dataset Mols as Training Set but with the parameters obtained from 2	27		5	0.95	0.19	0.47	0.41	-	-

[a]: model number; [b]: training set molecule number; [c]: test set molecule number; [d]: number of optimal principal components; [e]: determination coefficient; [f]: standard deviation of recalculation; [g]: cross-validated determination coefficient; [h]: standard deviation of internal prediction in cross-validation; [i]: predictive determination coefficient; [j]: standard deviation of external prediction

Table S20. Docking assessment results expressed as docking accuracies.

Redocking	Type[a]	Experimental[e]	Randomized[f]
Plants	PLP[c]	0.43	0.46
	PLP95[c]	0.46	0.50
	ChemPLP[c]	0.5	0.52
Smina	AD4_scoring[d]	0.30	0.31
	Vina[d]	0.54	0.54
	Vinardo[d]	0.65	0.69
Smina Min[b]	AD4_scoring	0.37	0.37
	Vina	0.50	0.50
	Vinardo	0.52	0.52

[a]: type of docking assessment; [b]: results after complex minimization; [c]: Plants' scoring functions; [d]: Smina's scoring functions; [e]: docking experiments starting from experimental bound conformations; [f]: docking experiments using randomized conformations

Table S21. Liquid chromatography–mass spectrometry (LC–MS) of synthetic peptides including an additional fluorescein (fluo) molecule. Name, elution retention time (tR), expected molecular weight (Da), calculated (cal.) and observed (obs.) molecular ions of each peptide are reported.

peptide	t_R (min)	mass (Da)	$[M+2H]^{2+}$		$[M+3H]^{3+}$		$[M+4H]^{4+}$		$[M+5H]^{5+}$	
			cal.	obs.	cal.	obs.	cal.	obs.	cal.	obs.
pHDAC4	7.5	1975.1	988.5	988.6	659.3	659.4	494.7	495.1	396.0	-
fluo-pHDAC4	10.2	2291.1	1146.5	1146.7	764.7	764.9	573.7	574.1	459.2	459.3

Table S22. List of 27 DNA decamer/ligand crystal complexes with associated pK_D.

PDB Code	pK_D	DNA	Name
**129D**	7.37	CGCGAATTCGCG	Pibenzimol
**1D43**	8.51	CGCGAATTCGCG	HOECHST 33258
**1D44**	8.51	CGCGAATTCGCG	HOECHST 33258
**1D45**	8.51	CGCGAATTCGCG	HOECHST 33258
**1D46**	8.51	CGCGAATTCGCG	HOECHST 33258
**1D63**	7.04	CGCAAATTTGCG	BERENIL
**1M6F**	7.85	CGCGAATTCGCG	CGP40215A
**1QV4**	6.91	CGTGAATTCACG	Methylproamine
**1QV8**	6.94	CGCGAATTCGCG	proamine
**1VZK**	8.45	CGCGAATTCGCG	DB818
**227D**	6.83	CGCGAATTCGCG	DB75
**2B0K**	8.43	CGCGAATTCGCG	DB921
**2I2I**	7.04	CGCGAATTCGCG	DB293
**2NLM**	7.08	CGCGAATTCGCG	DB911
**3U05**	8.68	CGCGAATTCGCG	DB1804
**3U08**	7.59	CGCGAATTCGCG	DB1963
**3U0U**	8.39	CGCGAATTCGCG	DB1883
**5T4W**	5.48	CGTGAATTCACG	DAPI
**7KU4**	8.82	CGCGAATTCGCG	DB818
**7KWK**	8.19	CGCGAATTCGCG	DB1879
**8EC1**	9.00	CGCGAATTCGCG	DB1476
**8ED6**	9.00	CGCGAATTCGCG	DB1476
**8EDA**	7.92	CGCGATATCGCG	DB1476
**8F1S**	8.70	CGCAAAAAAGCG	DB1476
**8F1V**	8.70	CGCAAAAAAGCG	DB1476
**8FDQ**	9.30	CGCAAATTTCGC	DB1476
**8FDR**	7.82	CGCGAAAAGCCG	DB1476
